# Outage Performance Analysis of Improper Gaussian Signaling for Two-User Downlink NOMA Systems with Imperfect Successive Interference Cancellation

**DOI:** 10.3390/e25081172

**Published:** 2023-08-06

**Authors:** Yaxuan Liu, Huadong Gao, Hao Cheng, Yili Xia, Wenjiang Pei

**Affiliations:** 1 School of Information Science and Engineering, Southeast University, Nanjing 210096, China; liuyaxuan@seu.edu.cn; 2 Guangdong Communications and Networks Institute, Guangzhou 510070, China; gaohd@gdcni.cn; 3 School of Internet of Things, Nanjing University of Posts and Telecommunications, Nanjing 210003, China; haocheng@njupt.edu.cn; 4 Frontiers Science Center for Mobile Information Communication and Security, Southeast University, Nanjing 210096, China

**Keywords:** improper Gaussian signaling (IGS), non-orthogonal multiple access (NOMA), imperfect SIC, outage probability, statistical CSI, power allocation

## Abstract

The improper Gaussian signaling (IGS) technique can improve the achievable rate of an interference-limited network by fully exploiting the second-order statistics of complex signaling. This paper addresses the outage performance analysis of a two-user downlink non-orthogonal multiple access (NOMA) system using the IGS technique in the presence of imperfect successive interference cancellation (SIC). The strong channel user (SU) adopts the IGS, while the weak channel user (WU) adopts the traditional proper Gaussian signaling (PGS). Considering a practical scenario where the transmitter has obtained the statistics of the channel coefficients instead of the instantaneous channel state information (CSI), the expressions of the achievable rates of both users under residual interference due to imperfect SIC are derived, together with their outage probabilities, subject to predetermined target rates and channel statistics. Given a fixed transmit power of the WU, both the transmit power and the degree of impropriety of the SU are optimized to minimize the outage probability subject to the outage constraint of the WU. Numerical results are provided to assess the benefits of the proposed IGS-based downlink NOMA system, which are consistent with the analysis.

## 1. Introduction

The fifth generation (5G) and beyond communication systems support numerous wireless devices and a variety of services. To meet the massive demand for data throughput, non-orthogonal multiple access (NOMA) has been proposed as a promising technology to improve the spectral efficiency of communication systems [1,2,3,4]. The existing NOMA schemes can be mainly divided into two categories: power domain multiplexing [5,6] and code domain multiplexing [7,8,9]. Power domain multiplexing means different users are allowed to transmit data using the same communication resources with different power levels according to their channel conditions, and the base station (BS) broadcasts the superposed signals for multiple users at different power levels. Typically, the BS allocates more power to the user with a weak channel strength. The weak channel user decodes its signal by treating the signal intended for the user with the strong channel strength as interference, and the strong channel user needs to first remove the signal for the weak channel user from the received signal through successive interference cancellation (SIC) [10] and then decode its own signal. Recently, several works have considered discrete signaling, which can be formed by practical modulation schemes without SIC [11,12]. The discrete signaling was adopted in a multi-user downlink NOMA system without SIC in [11], where the outage rates were very close to the outage capacity region given in [13]. Similarly, the discrete signaling without SIC achieves a rate close to Gaussian signaling with perfect SIC for the interference channel [12]. However, the assumption of Gaussian signaling is still common in the theoretical analysis of communication systems and is useful for its simplicity in mathematical manipulations, and SIC should be considered under the Gaussian signaling setting to characterize the capacity and achievable rate region of a NOMA system. In a real-world scenario, a perfect SIC is impractical because the interference cannot be canceled completely due to deep fading, imperfect decoding, channel estimation errors, and so on [10,14,15]. It has been discovered in [16] that the NOMA system performance converges to an error floor in the high signal-to-noise ratio (SNR) region with imperfect SIC and, thus, a zero diversity order is obtained. Therefore, it is of great interest to consider the detrimental impacts of residual interference due to the imperfect SIC on NOMA systems.

Mitigating interference in communication systems is a challenging research problem. It is well known that statistical signal characteristics significantly affect achievable rates and communication performance. Proper Gaussian signaling (PGS) is a common assumption in communication systems, where the real and imaginary parts of complex signals are uncorrelated zero-mean Gaussian random variables with equal power [17,18]. Most existing studies on communication systems have adopted the PGS assumption without any justification because it is known to achieve the maximum achievable rate in an additive white Gaussian noise channel [17]. However, by allowing the real and imaginary parts of the signals to be correlated or to have unequal powers, improper Gaussian signaling (IGS) can increase the achievable rates and enlarge the capacity regions in interference-limited scenarios, including interference channels [19,20], cognitive radio scenarios [21], relay networks [22,23], and so on. Fundamental studies on the second-order statistics of improper complex random vectors were performed in [24,25,26,27]. IGS was used to model the resultant signals from in-phase and quadrature imbalance in [28]. The usefulness of the IGS for interference alignment in wireless communications was investigated in [29]. In [30], maximally IGS was applied to a two-user single-input–single-output (SISO) interference channel to achieve a close-to-optimal sum rate in the interference-limited regime with strong interference and relatively weak noise. In [31], an achievable rate expression for a general multi-user multiple-input–multiple-output interference channel with IGS was derived, followed by the Pareto boundary characterization of the achievable rate region in a two-user SISO setting. The study was further extended to the multiple-input–single-output scenario in [32]. In [21], IGS was adopted to underlay cognitive radio networks to improve the achievable rate of the unlicensed secondary user. A similar cognitive radio scenario was studied in [33], where only statistical channel state information (CSI) was assumed to be available at the secondary user nodes, and IGS was shown to improve the outage performance of the secondary user while satisfying the quality-of-service (QoS) constraint of the primary user. The benefits of adopting IGS in a two-hop alternate relaying system were investigated in [22], where significant performance improvement can be achieved by IGS, especially when the first hop is a bottleneck.

Although research has been conducted to optimize IGS-based schemes in various wireless communication scenarios to improve the system throughput and reliability, the application of IGS in NOMA systems has just emerged. A two-user downlink NOMA system with imperfect SIC was considered in [34], where the closed-form expressions of the sum rates of both users using IGS were carried out. Assuming that the strong user and the weak user employ IGS and PGS, respectively, the degree of impropriety of the strong user was optimized to maximize the sum rate. The two-user NOMA system was further investigated in [35], where the strong user employs PGS, and the weak user adopts IGS. Optimization problems were formulated to maximize the rate of either the strong user or the weak user subject to its power and the impropriety degree of the weak user, assuming a fixed power and a predetermined QoS requirement of the other user. The sum rate was also maximized using a Q-learning-based algorithm. In [36], the outage probabilities and ergodic capacities were studied in a NOMA system with imperfect SIC, in which the strong user applies IGS, and a characterization of the system performance at high SNR was given. The fairness optimization problem was formulated to minimize the maximum outage probability of the two users by adjusting the power allocation factor and the circularity coefficient of the strong user. However, when different QoS constraints are imposed on the two users, other metrics should be considered.

In this paper, we investigate a downlink NOMA system with imperfect SIC, where a BS serves a strong channel user and a weak channel user. The strong user (SU) can employ either proper or improper signaling, while the weak user (WU) adopts proper signaling. Only the statistical CSI is assumed to be known at the BS, which is more practical than the knowledge of the instantaneous CSI assumed in [34,35]. We provide a solution to improve the performance of the SU while satisfying the QoS of the WU. The main contributions of this paper are summarized as follows:

- We derive the instantaneous rates of both users in the downlink NOMA system with imperfect SIC. Based on the rate expressions and the Rayleigh fading channel statistics, their outage probabilities are also derived in closed form.
- Unlike the previous attempt to optimize the fairness of both users [36], we consider a scenario where the two users have different requirements for data throughput and reliability. The performance optimization problem of the SU is formulated, where its power and degree of impropriety are adjusted to minimize its outage probability, subject to a predetermined target rate, while fulfilling the specific maximum outage probability constraint of the WU. The solution to the problem is derived, followed by an analysis of the condition for the IGS scheme to outperform the PGS one, which is mainly determined by the target rates of both users, the power of the signal intended for the WU, and the power ratio between two users.
- We investigate the outage probabilities of the SU in both PGS and IGS designs at extreme SNRs and find that the IGS can bring benefits at a high target rate of the WU and a large power ratio between the SU and WU. Through simulation, it is shown that the SU benefits from the IGS-based transmission strategy in a certain SNR range under certain conditions involving the target rates of both users and their power ratio.

The rest of this paper is organized as follows. Section 2 describes the two-user downlink NOMA system model. Section 3 derives the outage probabilities for both users in the NOMA system. In Section 4, the signal parameters of the SU are adjusted to minimize its outage probability under both PGS and IGS constraints while guaranteeing the QoS of the WU, along with a brief characterization of the system performance at low and high SNRs. Numerical experiments are then conducted to investigate the outage probabilities of both users in Section 5. Finally, we conclude the paper in Section 6.

The notations that will be used throughout the paper are listed and explained in Table 1.

## 2. System Model

### 2.1. Preliminaries for Improper Random Variables

For a zero-mean complex random variable *x*, its variance $\sigma_{x}^{2}$ and complementary variance ${\tilde{\sigma }}_{x}^{2}$ are, respectively, defined as [17]

(1) $$\sigma_{x}^{2}=E\left\{{\left|x\right|}^{2}\right\},\;{\tilde{\sigma }}_{x}^{2}=E\left\{x^{2}\right\},$$

where E{·} denotes the statistical expectation. It is easy to verify that $\sigma_{x}^{2}\geq 0$ and $|{\tilde{\sigma }}_{x}^{2}|\leq \sigma_{x}^{2}$, which is the sufficient and necessary condition for the variance $\sigma_{x}^{2}$ and complementary variance ${\tilde{\sigma }}_{x}^{2}$ to be a valid pair. The impropriety degree, or circularity coefficient of *x*, is defined as

(2) $$\kappa_{x}=\frac{|{\tilde{\sigma }}_{x}^{2}|}{\sigma_{x}^{2}}.$$

Obviously, we have $0\leq \kappa_{x}\leq 1$. The random variable *x* is called proper if ${\tilde{\sigma }}_{x}^{2}=0$, i.e., $\kappa_{x}=0$; otherwise, it is called improper. Specifically, *x* is called maximally improper if $\kappa_{x}=1$. Furthermore, if *x* is a Gaussian variable, then the differential entropy of *x* is a function of both its variance and complementary variance, given by [18]

(3) $$h\left(x\right)=\frac{1}{2}\log_{2}\left(\pi e\left(\sigma_{x}^{4}-{\left|{\tilde{\sigma }}_{x}^{2}\right|}^{2}\right)\right)=\frac{1}{2}\log_{2}\left(\pi e\sigma_{x}^{4}\left(1-\kappa_{x}^{2}\right)\right).$$

The interested readers are referred to [18,25,37] for a comprehensive treatment of the topic.

### 2.2. System Description

**Figure 1:**
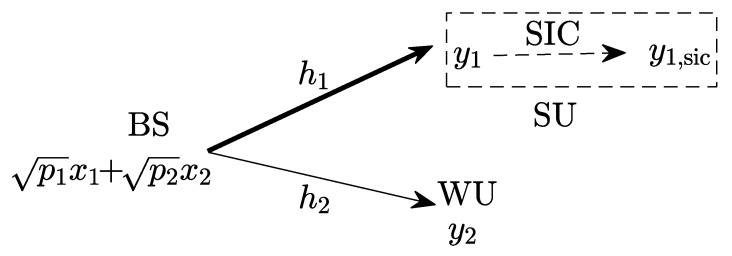
Two-user downlink non-orthogonal multiple access (NOMA) system model.

Consider a downlink NOMA system where a single-antenna base station (BS) sends independent information to two single-antenna receivers, including a strong channel user (SU, user 1) and a weak channel user (WU, user 2), as shown in Figure 1. The channel condition of the SU is satisfactory, but that of the WU is significantly degraded.

The received signal of the SU and WU can, respectively, be expressed as

(4) $$\begin{array}{cc} y_{1}=\; & \sqrt{p_{2}}h_{1}x_{2}+\underset{z_{12}}{\underset{︸}{\sqrt{p_{1}}h_{1}x_{1}+n_{1}}}, \end{array}$$

(5) $$\begin{array}{cc} y_{2}=\; & \sqrt{p_{2}}h_{2}x_{2}+\underset{z_{2}}{\underset{︸}{\sqrt{p_{1}}h_{2}x_{1}+n_{2}}}, \end{array}$$

where $h_{i}$ denotes the channel coefficient from the BS to user i,i=1,2, modeled as a Rayleigh fading channel; $n_{i}$ represents the noise at the receiver end of user *i*, modeled as a zero-mean white proper Gaussian additive random variable with variance $\sigma_{n_{i}}^{2}$; $p_{i}$ is the allocated power of user *i*, and $x_{i}$ is the transmitted signal intended for user *i* with zero mean and unit variance. According to the NOMA principle, the WU should be allocated with a higher power than the SU, i.e., $p_{1}\leq p_{2}$, so that the WU can decode its signal directly by treating the signal of the SU as interference, and the term $z_{2}$ in (5) denotes the interference-plus-noise component of the WU. As for the SU, it first decodes the signal of the WU and subtracts it from the received signal $y_{1}$ by performing SIC, and then detects its own signal. The term $z_{12}$ in (4) denotes the interference-plus-noise component to decode the signal of the WU at the SU end. In practice, the SIC detector at the SU is likely to be imperfect, so there is a residual interference component after decoding. The received signal of the SU after SIC can be modeled as [34]

(6) $$y_{1,\mathrm{sic}}=\sqrt{p_{1}}h_{1}x_{1}+\underset{z_{1}}{\underset{︸}{\sqrt{p_{2}}h_{r}x_{2}+n_{1}}},$$

where $h_{r}$ represents the residual interference channel coefficient for the imperfect SIC at the SU, and $z_{1}$ is the interference-plus-noise component to decode its own signal after SIC.

By the maximum entropy theorem, the PGS can achieve the maximum achievable rate in an additive white Gaussian noise channel [17]. Since the channel condition of the WU has been severely degraded, but the channel condition of the SU makes its QoS requirement easy to fulfill, we assume that the WU uses PGS, and the SU can adopt either PGS or IGS according to its power constraints. Therefore, $x_{1}$ in (4)–(6) is an improper Gaussian signal with its impropriety degree denoted as κ, and $x_{2}$ is proper. Since both $x_{1}$ and $x_{2}$ have unit variance, i.e., $\sigma_{x_{1}}^{2}=\sigma_{x_{2}}^{2}=1$, their complementary variances obey $|{\tilde{\sigma }}_{x_{1}}^{2}|=\kappa$ and ${\tilde{\sigma }}_{x_{2}}^{2}=0$, respectively. Based on the analysis in [31], for a received signal *y* and an interference-plus-noise signal *z*, the achievable rate is given by

(7) $$R=h\left(y\right)-h\left(z\right)=\frac{1}{2}\log_{2}\frac{\sigma_{y}^{4}-{\left|{\tilde{\sigma }}_{y}^{2}\right|}^{2}}{\sigma_{z}^{4}-{\left|{\tilde{\sigma }}_{z}^{2}\right|}^{2}}.$$

According to (5), the variances and complementary variances of $y_{2}$ and $z_{2}$ can be, respectively, given by

(8) $$\begin{array}{cc} \sigma_{y_{2}}^{2}\; & =p_{2}{\left|h_{2}\right|}^{2}+p_{1}{\left|h_{2}\right|}^{2}+\sigma_{n_{2}}^{2},\\ \sigma_{z_{2}}^{2}\; & =p_{1}{\left|h_{2}\right|}^{2}+\sigma_{n_{2}}^{2},\\ \left|{\tilde{\sigma }}_{y_{2}}^{2}\right|\; & =\left|{\tilde{\sigma }}_{z_{2}}^{2}\right|=p_{1}{\left|h_{2}\right|}^{2}\kappa . \end{array}$$

Upon substituting (8) into (7), the achievable rate of the WU can be given by

(9) $$R_{2}=h\left(y_{2}\right)-h\left(z_{2}\right)=\frac{1}{2}\log_{2}\left(1+\frac{p_{2}^{2}\gamma_{2}^{2}+2p_{2}\gamma_{2}\left(p_{1}\gamma_{2}+1\right)}{{\left(p_{1}\gamma_{2}+1\right)}^{2}-p_{1}^{2}\gamma_{2}^{2}\kappa^{2}}\right),$$

where $\gamma_{2}={\left|h_{2}\right|}^{2}/\sigma_{n_{2}}^{2}$ is the instantaneous channel-to-noise ratio (CNR) of the WU. Similarly, according to (4), the variances and complementary variances of $y_{1}$ and $z_{12}$ can, respectively, be given by

(10) $$\begin{array}{cc} \sigma_{y_{1}}^{2} & =p_{2}{\left|h_{1}\right|}^{2}+p_{1}{\left|h_{1}\right|}^{2}+\sigma_{n_{1}}^{2},\\ \sigma_{z_{12}}^{2} & =p_{1}{\left|h_{1}\right|}^{2}+\sigma_{n_{1}}^{2},\\ \left|{\tilde{\sigma }}_{y_{1}}^{2}\right| & =\left|{\tilde{\sigma }}_{z_{12}}^{2}\right|=p_{1}{\left|h_{1}\right|}^{2}\kappa , \end{array}$$

and the achievable rate to decode the signal of the WU at the SU end is given by

(11) $$R_{12}=h\left(y_{1}\right)-h\left(z_{12}\right)=\frac{1}{2}\log_{2}\left(1+\frac{p_{2}^{2}\gamma_{1}^{2}+2p_{2}\gamma_{1}\left(p_{1}\gamma_{1}+1\right)}{{\left(p_{1}\gamma_{1}+1\right)}^{2}-p_{1}^{2}\gamma_{1}^{2}\kappa^{2}}\right),$$

where $\gamma_{1}={\left|h_{1}\right|}^{2}/\sigma_{n_{1}}^{2}$ is the instantaneous CNR of the SU. It can also be obtained from (6) that

(12) $$\begin{array}{cc} \sigma_{y_{1,\mathrm{sic}}}^{2}\; & =p_{1}{\left|h_{1}\right|}^{2}+p_{2}{\left|h_{r}\right|}^{2}+\sigma_{n_{1}}^{2},\\ \sigma_{z_{1}}^{2}\; & =p_{2}{\left|h_{r}\right|}^{2}+\sigma_{n_{1}}^{2},\\ \left|{\tilde{\sigma }}_{y_{1,\mathrm{sic}}}^{2}\right|\; & =p_{1}{\left|h_{1}\right|}^{2}\kappa ,\\ {\tilde{\sigma }}_{z_{1}}^{2}\; & =0, \end{array}$$

and the achievable rate of the SU when decoding its own signal after SIC is given by

(13) $$R_{1}=h\left(y_{1,\mathrm{sic}}\right)-h\left(z_{1}\right)=\frac{1}{2}\log_{2}\left(1+\frac{p_{1}^{2}\gamma_{1}^{2}\left(1-\kappa^{2}\right)+2p_{1}\gamma_{1}\left(p_{2}\gamma_{r}+1\right)}{{\left(p_{2}\gamma_{r}+1\right)}^{2}}\right)$$

according to (10) and (7), where $\gamma_{r}={\left|h_{r}\right|}^{2}/\sigma_{n_{1}}^{2}$ is the instantaneous CNR of the residual interference channel.

## 3. Outage Probability Analysis

In practice, since the instantaneous CSI may be unavailable at the BS, owing to the lack of uplink feedback resources, only the statistical CSI of both links is available. The channel coefficients from the BS to user i,i=1,2, i.e., $h_{i}$, can be modeled as independent zero-mean proper Gaussian random variables; thus, the instantaneous CSI $\gamma_{i}={\left|h_{i}\right|}^{2}/\sigma_{n_{i}}^{2}$ are exponential random variables with means $E\left\{\gamma_{i}\right\}=\sigma_{h_{i}}^{2}/\sigma_{n_{i}}^{2}≜{\bar{\gamma }}_{i}$. The cumulative distribution functions (CDF) and probability density functions of $\gamma_{i}$ are, respectively, given by

(14) $$\begin{array}{cccc} & F_{\gamma_{i}}\left(\gamma \right)=\mathrm{Pr}\left[\gamma_{i}<\gamma \right]=1-\exp\left(-\frac{\gamma }{{\bar{\gamma }}_{i}}\right), & \; & \;\gamma >0,\\ \; & p_{\gamma_{i}}\left(\gamma \right)=\frac{d}{d\gamma }F_{\gamma_{i}}\left(\gamma \right)=\frac{1}{{\bar{\gamma }}_{i}}\exp\left(-\frac{\gamma }{{\bar{\gamma }}_{i}}\right), & \; & \;\gamma >0, \end{array}$$

where Pr[·] denotes the probability. The average CNR of the SU is greater than that of the WU, i.e., ${\bar{\gamma }}_{1}\geq {\bar{\gamma }}_{2}$. The residual interference channel CNR $\gamma_{r}$ can also be modeled as an exponential random variable with a mean of $E\left\{\gamma_{r}\right\}=\sigma_{h_{r}}^{2}/\sigma_{n_{1}}^{2}=\xi {\bar{\gamma }}_{1}$, where ξ represents the level of residual interference [36,38,39,40,41]. The CDF of $\gamma_{r}$ is given by

(15) $$F_{\gamma_{r}}\left(\gamma_{r}\right)=\mathrm{Pr}\left[\gamma_{r}<\gamma \right]=\left\{\begin{array}{cc} 1-\exp\left(-\frac{\gamma }{\xi {\bar{\gamma }}_{1}}\right), & \gamma >0,\\ 0, & \gamma \leq 0. \end{array}\right.$$

The case ξ=0 refers to perfect SIC, and ξ=1 means fully imperfect SIC. In what follows, the overall outage probability of the proposed downlink NOMA system employing IGS is investigated to assess its error performance.

### 3.1. Weak User Outage Probability

Let $R_{0,i}$ be the target rate of user i,i=1,2. The outage probability of the WU, denoted by $P_{\mathrm{out},2}$, is defined as

(16) $$P_{\mathrm{out},2}=\mathrm{Pr}\left[R_{2}<R_{0,2}\right].$$

Substituting (9) into (16), we have

(17) $$\begin{array}{cc} P_{\mathrm{out},2}= & \mathrm{Pr}\left[\frac{p_{2}^{2}\gamma_{2}^{2}+2p_{2}\gamma_{2}\left(p_{1}\gamma_{2}+1\right)}{{\left(p_{1}\gamma_{2}+1\right)}^{2}-p_{1}^{2}\gamma_{2}^{2}\kappa^{2}}<\Gamma_{2}\right]\\ = & \mathrm{Pr}\left[\left(\gamma_{2}^{-1}+p_{2}{\tilde{v}}_{2}\right)\left(\gamma_{2}^{-1}-p_{2}v_{2}\right)>0\right], \end{array}$$

where $\Gamma_{2}=2^{2R_{0,2}}-1$,

(18) $${\tilde{v}}_{2}=\frac{\sqrt{p_{2}^{2}\left(1+\Gamma_{2}\right)+\Gamma_{2}^{2}p_{1}^{2}\kappa^{2}}-p_{2}+\Gamma_{2}p_{1}}{p_{2}\Gamma_{2}}>0,$$

(19) $$v_{2}=\frac{\sqrt{p_{2}^{2}\left(1+\Gamma_{2}\right)+\Gamma_{2}^{2}p_{1}^{2}\kappa^{2}}+p_{2}-\Gamma_{2}p_{1}}{p_{2}\Gamma_{2}}.$$

Since $\gamma_{2}^{-1}+p_{2}{\tilde{v}}_{2}>0$, if $v_{2}>0$, according to (14), the outage probability of the WU in (17) can be rewritten as

(20) $$P_{\mathrm{out},2}=\mathrm{Pr}\left[\gamma_{2}<\frac{1}{p_{2}v_{2}}\right]=1-\exp\left(-\frac{1}{{\bar{\gamma }}_{2}p_{2}v_{2}}\right).$$

Otherwise, if $v_{2}\leq 0$, then the inequality inside the probability in (17) holds as long as $\gamma_{2}>0$, to yield $P_{\mathrm{out},2}=1$. Overall, the outage probability of the WU is given by

(21) $$P_{\mathrm{out},2}=\left\{\begin{array}{cc} 1-\exp\left(-\frac{1}{{\bar{\gamma }}_{2}p_{2}v_{2}}\right), & v_{2}>0,\\ 1, & v_{2}\leq 0, \end{array}\right.$$

where $v_{2}$ is given in (19).

The rest of this paper only considers the case when $P_{\mathrm{out},2}<1$, or equivalently, $v_{2}>0$ holds, so that the outage does not always occur.

### 3.2. Strong User Outage Probability

An outage occurs at the SU end when the achievable rate to decode its signal is lower than its target rate $R_{0,1}$, or the rate to decode the signal of the WU is lower than the target rate $R_{0,2}$ of the WU. The outage probability of the SU is given by

(22) $$P_{\mathrm{out},1}=\mathrm{Pr}\left[R_{12}<R_{0,2}\cup R_{1}<R_{0,1}\right].$$

Upon substituting (11) and (13) into (22), we have

(23) $$\begin{array}{cc} P_{\mathrm{out},1}=\; & \mathrm{Pr}\left[\frac{p_{2}^{2}\gamma_{1}^{2}+2p_{2}\gamma_{1}\left(p_{1}\gamma_{1}+1\right)}{{\left(p_{1}\gamma_{1}+1\right)}^{2}-p_{1}^{2}\gamma_{1}^{2}\kappa^{2}}<\Gamma_{2}\right.\\ \; & \;\left.\cup \;\frac{p_{1}^{2}\gamma_{1}^{2}\left(1-\kappa^{2}\right)+2p_{1}\gamma_{1}\left(p_{2}\gamma_{r}+1\right)}{{\left(p_{2}\gamma_{r}+1\right)}^{2}}<\Gamma_{1}\right], \end{array}$$

where $\Gamma_{1}=2^{2R_{0,1}}-1$. Similar to the analysis in Section 3.1, when $v_{2}>0$, the first inequality in (23) holds if and only if $\gamma_{1}<1/\left(p_{2}v_{2}\right)$. Note that both $\gamma_{1}$ and $\gamma_{r}$ are in the second inequality in (23). Given a fixed $\gamma_{1}$, this inequality can be rewritten as

(24) $$\gamma_{r}>\gamma_{r,0}\left(\gamma_{1}\right)≜v_{1}\gamma_{1}-\frac{1}{p_{2}},$$

where

(25) $$v_{1}≜\frac{p_{1}\left[1+\sqrt{1+\Gamma_{1}\left(1-\kappa^{2}\right)}\right]}{p_{2}\Gamma_{1}}.$$

Therefore, $P_{\mathrm{out},1}$ is given by

(26) $$\begin{array}{cc} P_{\mathrm{out},1}=\; & \mathrm{Pr}\left[\gamma_{1}<\frac{1}{p_{2}v_{2}}\;\cup \;\gamma_{r}>\gamma_{r,0}\left(\gamma_{1}\right)\right]\\ =\; & \mathrm{Pr}\left[\gamma_{1}<\frac{1}{p_{2}v_{2}}\right]+\mathrm{Pr}\left[\gamma_{1}\geq \frac{1}{p_{2}v_{2}}\;\cap \;\gamma_{r}>\gamma_{r,0}\left(\gamma_{1}\right)\right]\\ =\; & F_{\gamma_{1}}\left(\frac{1}{p_{2}v_{2}}\right)+\int_{\frac{1}{p_{2}v_{2}}}^{\infty }\mathrm{Pr}\left[\gamma_{r}>\gamma_{r,0}\left(\gamma_{1}\right)|\gamma_{1}\right]p_{\gamma_{1}}\left(\gamma_{1}\right)d\gamma_{1}\\ =\; & F_{\gamma_{1}}\left(\frac{1}{p_{2}v_{2}}\right)+\underset{E}{\underset{︸}{\int_{\frac{1}{p_{2}v_{2}}}^{\infty }\left[1-F_{\gamma_{r}}\left(\gamma_{r,0}\left(\gamma_{1}\right)\right)\right]p_{\gamma_{1}}\left(\gamma_{1}\right)d\gamma_{1}}}. \end{array}$$

According to (14) and (15), function *E* in (26) can be rewritten as

(27) $$E=\left\{\begin{array}{cc} \frac{1}{{\bar{\gamma }}_{1}}\int_{\frac{1}{p_{2}v_{2}}}^{\infty }\exp\left(-\frac{\gamma_{r,0}\left(\gamma_{1}\right)}{\xi {\bar{\gamma }}_{1}}-\frac{\gamma_{1}}{{\bar{\gamma }}_{1}}\right)d\gamma_{1}, & v_{1}>v_{2},\\ F_{\gamma_{1}}\left(\frac{1}{p_{2}v_{1}}\right)-F_{\gamma_{1}}\left(\frac{1}{p_{2}v_{2}}\right)\\ \;+\frac{1}{{\bar{\gamma }}_{1}}\int_{\frac{1}{p_{2}v_{1}}}^{\infty }\exp\left(-\frac{\gamma_{r,0}\left(\gamma_{1}\right)}{\xi {\bar{\gamma }}_{1}}-\frac{\gamma_{1}}{{\bar{\gamma }}_{1}}\right)d\gamma_{1}, & v_{1}\leq v_{2}. \end{array}\right.$$

Upon combining (14), (24), (26), and (27), it is not difficult to verify that the SU outage probability $P_{\mathrm{out},1}$ can be further expressed as

(28) $$P_{\mathrm{out},1}=\left\{\begin{array}{cc} 1-\exp\left(-\frac{1}{{\bar{\gamma }}_{1}p_{2}v_{2}}\right)\left[1-\frac{\exp\left[-\frac{1}{p_{2}\xi {\bar{\gamma }}_{1}}\left(\frac{v_{1}}{v_{2}}-1\right)\right]}{\frac{v_{1}}{\xi }+1}\right], & v_{1}>v_{2},\\ 1-\exp\left(-\frac{1}{{\bar{\gamma }}_{1}p_{2}v_{1}}\right)\left[1-\frac{1}{\frac{v_{1}}{\xi }+1}\right], & v_{1}\leq v_{2}, \end{array}\right.$$

where $v_{1}$ and $v_{2}$ are functions of $p_{1}$, $p_{2}$, and κ, given in (25) and (19), respectively.

## 4. Optimal Signaling Design

We next study the outage performance optimization of the SU by jointly optimizing its power $p_{1}$ and impropriety degree κ, subject to a predetermined QoS of the WU represented by a maximum outage probability threshold $P_{\mathrm{out},\mathrm{th}}$ for a target rate $R_{0,2}$. The outage performance optimization problem can be formulated as

$$\begin{array}{cc} \min_{p_{1},\kappa }\; & P_{\mathrm{out},1} \end{array}$$

(29a) $$\begin{array}{cc} \mathrm{subject}\;\mathrm{to}\; & P_{\mathrm{out},2}\leq P_{\mathrm{out},\mathrm{th}}, \end{array}$$

(29b) $$\begin{array}{cc} \; & 0<p_{1}\leq p_{1,\max}, \end{array}$$

(29c) $$\begin{array}{cc} \; & 0\leq \kappa \leq 1, \end{array}$$

(29d) $$\begin{array}{cc} \; & v_{2}>0, \end{array}$$

where $p_{1,\max}$ represents the maximum power budget of the SU. According to our system model, the WU is allocated with more power than the SU, so $p_{1,\max}$ should not be greater than $p_{2}$. However, we will ignore this constraint while solving the optimization (29) in order to show that our proposed optimal signaling design still works when $p_{1}>p_{2}$. In the PGS design, the impropriety degree κ of the SU is always zero, so its power $p_{1}$ should be optimized subject to (29a,b,d).

According to (28), $P_{\mathrm{out},1}$ can be viewed as a function of $v_{1}$ and $v_{2}$ with the following properties:

- Given a fixed $v_{2}$, $P_{\mathrm{out},1}$ decreases in $v_{1}$.
- Given a fixed $v_{1}$, $P_{\mathrm{out},1}$ decreases in $v_{2}$ when $v_{2}<v_{1}$ and remains static when $v_{2}\geq v_{1}$. Note that when $v_{2}<v_{1}$, the partial derivative of $P_{\mathrm{out},1}$ with respect to $v_{2}$ is
  (30) $$\frac{\partial P_{\mathrm{out},1}}{\partial v_{2}}=-\frac{1}{{\bar{\gamma }}_{1}p_{2}v_{2}^{2}}\exp\left(-\frac{1}{{\bar{\gamma }}_{1}p_{2}v_{2}}\right)\left[1-\exp\left[-\frac{1}{p_{2}\xi {\bar{\gamma }}_{1}}\left(\frac{v_{1}}{v_{2}}-1\right)\right]\right]<0.$$

Upon considering the expression of $P_{\mathrm{out},2}$ in (21) together, it is obvious that a greater $v_{2}$ may improve the outage performance of both the SU and the WU, and a greater $v_{1}$ can improve the outage performance of the SU while maintaining the outage probability of the WU.

Upon replacing $P_{\mathrm{out},2}$ in (29a) with the expression in (21), we have

(31) $$v_{2}\geq v_{2,\mathrm{th}}≜-\frac{1}{{\bar{\gamma }}_{2}p_{2}\log\left(1-P_{\mathrm{out},\mathrm{th}}\right)}.$$

According to (25), we have $v_{1}>0$, and

(32) $$\kappa^{2}=1-\frac{p_{2}}{p_{1}}v_{1}\left(\frac{p_{2}\Gamma_{1}}{p_{1}}v_{1}-2\right).$$

Substituting (32) into (19) yields $p_{1}=\lambda \left(v_{1},v_{2}\right)p_{2}$, where

(33) $$\lambda \left(v_{1},v_{2}\right)≜\frac{\Gamma_{1}v_{1}^{2}+{\left(v_{2}-\frac{1}{\Gamma_{2}}\right)}^{2}-\frac{1+\Gamma_{2}}{\Gamma_{2}^{2}}}{2\left(v_{1}-v_{2}+\frac{1}{\Gamma_{2}}\right)}.$$

Therefore, $p_{1}$ and $\kappa^{2}$ can, respectively, be expressed as

(34) $$\left\{\begin{array}{c} p_{1}=\lambda \left(v_{1},v_{2}\right)p_{2},\\ \kappa^{2}=1-\frac{v_{1}}{\lambda \left(v_{1},v_{2}\right)}\left(\frac{\Gamma_{1}v_{1}}{\lambda \left(v_{1},v_{2}\right)}-2\right). \end{array}\right.$$

Substituting (34) into conditions (29b,c) yields

(35) $$\begin{array}{cc} \kappa^{2}\geq 0\Rightarrow \; & \lambda \left(v_{1},v_{2}\right)\geq \left(\sqrt{1+\Gamma_{1}}-1\right)v_{1},\\ \kappa \leq 1\Rightarrow \; & \lambda \left(v_{1},v_{2}\right)\leq \frac{\Gamma_{1}v_{1}}{2},\\ p_{1}\leq p_{1,\max}\Rightarrow \; & \lambda \left(v_{1},v_{2}\right)\leq \lambda_{m}, \end{array}$$

where $\lambda_{m}=p_{1,\max}/p_{2}$. To ease the analysis, denote $v_{2}^{p}\left(v_{1}\right)$, $v_{2}^{i}\left(v_{1}\right)$, and $v_{2}^{m}\left(v_{1}\right)$ as the values of $v_{2}$ that make both sides of the inequalities in (35) equal, respectively, given by

(36) $$\begin{array}{cc} v_{2}^{p}\left(v_{1}\right)=\; & \frac{1}{\sqrt{1+\Gamma_{2}}-1}-\left(\sqrt{1+\Gamma_{1}}-1\right)v_{1},\\ v_{2}^{i}\left(v_{1}\right)=\; & \sqrt{\frac{1+\Gamma_{2}}{\Gamma_{2}^{2}}+\frac{\Gamma_{1}^{2}v_{1}^{2}}{4}}-\frac{\Gamma_{1}v_{1}}{2}+\frac{1}{\Gamma_{2}},\\ v_{2}^{m}\left(v_{1}\right)=\; & \sqrt{\lambda_{m}^{2}+2\lambda_{m}v_{1}-\Gamma_{1}v_{1}^{2}+\frac{1+\Gamma_{2}}{\Gamma_{2}^{2}}}+\frac{1}{\Gamma_{2}}-\lambda_{m}. \end{array}$$

Both $v_{2}^{p}\left(v_{1}\right)$ and $v_{2}^{i}\left(v_{1}\right)$ decrease in $v_{1}$. The function $v_{2}^{m}\left(v_{1}\right)$ increases in $v_{1}$ when $0<v_{1}\leq \lambda_{m}/\Gamma_{1}$ and decreases in $v_{1}$ when $v_{1}\geq \lambda_{m}/\Gamma_{1}$. Next, the following cases are considered to simplify the conditions in (35):

1. When $v_{1}-v_{2}+1/\Gamma_{2}=0$, the numerator on the right-hand side of (33) must be zero. After a few mathematical manipulations, we have
  (37) $$\begin{array}{cc} v_{1}=\; & v_{1}^{c}≜\frac{\sqrt{1+\Gamma_{2}}}{\Gamma_{2}\sqrt{1+\Gamma_{1}}},\\ v_{2}=\; & v_{2}^{c}≜\frac{1}{\Gamma_{2}}\left(1+\frac{\sqrt{1+\Gamma_{2}}}{\sqrt{1+\Gamma_{1}}}\right), \end{array}$$
  and
  (38) $$p_{1}=\frac{v_{1}^{c}p_{2}\Gamma_{1}}{1+\sqrt{1+\Gamma_{1}\left(1-\kappa^{2}\right)}},$$
  where κ can be assigned any value under the conditions in (29b,c). Note that $p_{1}$ given in (38) is increasing in κ, so that when κ=0, $p_{1}$ reaches its minimum $v_{1}^{c}p_{2}\left(\sqrt{1+\Gamma_{1}}-1\right)$, which has to be no greater than $p_{1,\max}$, and thus $v_{1}^{c}\leq v_{1}^{\mathrm{pm}}$, in which
  (39) $$v_{1}^{\mathrm{pm}}=\frac{\lambda_{m}}{\sqrt{1+\Gamma_{1}}-1}$$
  is the value of $v_{1}$ at $p_{1}=p_{1,\max}$ and κ=0, to give $v_{2}^{p}\left(v_{1}^{\mathrm{pm}}\right)=v_{2}^{m}\left(v_{1}^{\mathrm{pm}}\right)$. We prefer to let κ=0 in order to achieve the lowest power of the signal of the SU. Moreover, it is not difficult to verify that
  (40) $$v_{2}^{c}=v_{2}^{p}\left(v_{1}^{c}\right)=v_{2}^{i}\left(v_{1}^{c}\right)=v_{2}^{m}\left(v_{1}^{c}\right).$$
2. When $v_{1}-v_{2}+1/\Gamma_{2}<0$, according to (35), we have
  (41) $$\begin{array}{cc} \kappa^{2}\geq 0\Rightarrow & v_{2}\leq v_{2}^{p}\left(v_{1}\right),\\ \kappa \leq 1\Rightarrow & v_{2}\geq v_{2}^{i}\left(v_{1}\right),\\ p_{1}\leq p_{1,\max}\Rightarrow & v_{2}\geq v_{2}^{m}\left(v_{1}\right). \end{array}$$
  Both $v_{2}^{p}\left(\cdot \right)$ and $v_{2}^{i}\left(\cdot \right)$ are decreasing functions according to (36), and $v_{1}^{c}-v_{2}^{c}+1/\Gamma_{2}=0$ according to (37), to give $v_{1}<v_{1}^{c}$ and $v_{2}>v_{2}^{c}$. According to (36), the inequality $v_{2}^{p}\left(v_{1}\right)>v_{2}^{i}\left(v_{1}\right)$ holds as long as $0<v_{1}<v_{1}^{c}$. Since the inequality $v_{2}^{p}\left(v_{1}\right)\geq v_{2}^{m}\left(v_{1}\right)$ can be equivalently written as $\left(v_{1}-v_{1}^{c}\right)\left(v_{1}-v_{1}^{\mathrm{pm}}\right)\geq 0$, $v_{1}$ lies in the interval $\left(0,v_{1}^{\mathrm{pm}}\right]\cap \left(0,v_{1}^{c}\right)$.
3. When $v_{1}-v_{2}+1/\Gamma_{2}>0$, according to (35), we have
  (42) $$\begin{array}{cc} \kappa^{2}\geq 0\Rightarrow \; & v_{2}\geq v_{2}^{p}\left(v_{1}\right),\\ \kappa \leq 1\Rightarrow \; & v_{2}\leq v_{2}^{i}\left(v_{1}\right),\\ p_{1}\leq p_{1,\max}\Rightarrow \; & v_{2}\leq v_{2}^{m}\left(v_{1}\right). \end{array}$$
  Similar to the analysis in case 2, $v_{1}$ and $v_{2}$ satisfy $v_{1}>v_{1}^{c}$ and $v_{2}<v_{2}^{c}$, the inequality $v_{2}^{p}\left(v_{1}\right)<v_{2}^{i}\left(v_{1}\right)$ holds for any $v_{1}>v_{1}^{c}$, and $v_{2}^{p}\left(v_{1}\right)\leq v_{2}^{m}\left(v_{1}\right)$, as long as $0\leq v_{1}\leq v_{1}^{\mathrm{pm}}$. Hence, $v_{1}\in \left(v_{1}^{c},v_{1}^{\mathrm{pm}}\right]$.

**Figure 2:**
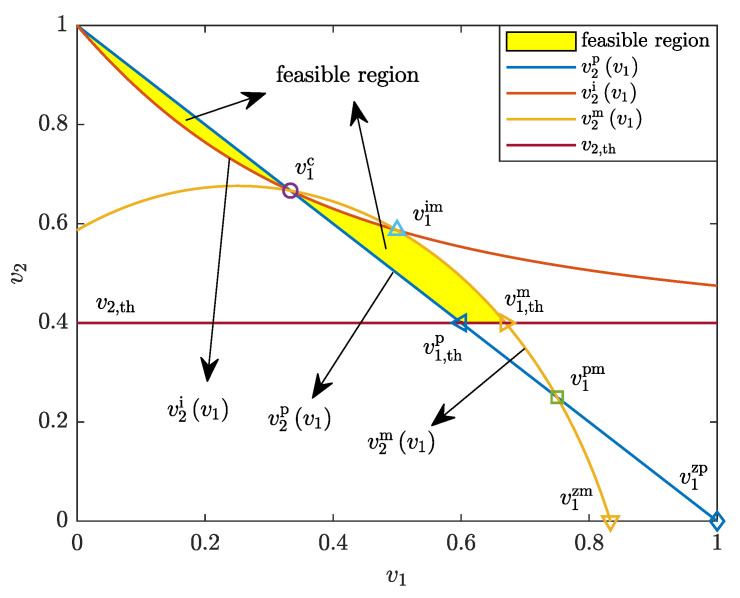
Feasible region of $v_{1}$ and $v_{2}$ with $R_{1}=1$ bps/Hz, $R_{2}=1$ bps/Hz, $\lambda_{m}=0.75$, and $v_{2,\mathrm{th}}=0.4$.

In summary, the conditions in (29) can be equivalently rewritten as

(43) $$\left\{\begin{array}{c} v_{2}^{L}\left(v_{1}\right)\leq v_{2}\leq v_{2}^{U}\left(v_{1}\right),\\ v_{2}\geq v_{2,\mathrm{th}},\\ 0<v_{1}\leq v_{1}^{\mathrm{pm}}, \end{array}\right.$$

where $v_{2}^{L}\left(\cdot \right)$ and $v_{2}^{U}\left(\cdot \right)$ are, respectively, given by

(44) $$v_{2}^{L}\left(v_{1}\right)=\left\{\begin{array}{cc} \max\left\{v_{2}^{i}\left(v_{1}\right),v_{2}^{m}\left(v_{1}\right)\right\}, & v_{1}\leq v_{1}^{c},\\ v_{2}^{p}\left(v_{1}\right), & v_{1}>v_{1}^{c}, \end{array}\right.$$

(45) $$\begin{array}{cc} v_{2}^{U}\left(v_{1}\right)\; & =\left\{\begin{array}{cc} v_{2}^{p}\left(v_{1}\right), & v_{1}\leq v_{1}^{c},\\ \min\left\{v_{2}^{i}\left(v_{1}\right),v_{2}^{m}\left(v_{1}\right)\right\}, & v_{1}>v_{1}^{c}, \end{array}\right.\\ \; & =\left\{\begin{array}{cc} v_{2}^{p}\left(v_{1}\right), & v_{1}\leq v_{1}^{c},\\ v_{2}^{i}\left(v_{1}\right), & v_{1}^{c}<v_{1}\leq v_{1}^{\mathrm{im}},\\ v_{2}^{m}\left(v_{1}\right), & v_{1}>\max\left\{v_{1}^{c},v_{1}^{\mathrm{im}}\right\}, \end{array}\right. \end{array}$$

and

(46) $$v_{1}^{\mathrm{im}}=\frac{2\lambda_{m}}{\Gamma_{1}}$$

is the value of $v_{1}$ at $p_{1}=p_{1,\max}$ and κ=1, making $v_{2}^{i}\left(v_{1}^{\mathrm{im}}\right)=v_{2}^{m}\left(v_{1}^{\mathrm{im}}\right)$. Since $v_{2}^{U}\left(v_{1}\right)<v_{2}^{U}\left(0\right)={\left(\sqrt{1+\Gamma_{2}}-1\right)}^{-1}$ for each $v_{1}>0$, $v_{2,\mathrm{th}}$ must be smaller than ${\left(\sqrt{1+\Gamma_{2}}-1\right)}^{-1}$, to give $p_{2}>-\left(\sqrt{1+\Gamma_{2}}-1\right)/\left[{\bar{\gamma }}_{2}\log\left(1-P_{\mathrm{out},\mathrm{th}}\right)\right]$. Figure 2 plots the feasible region of $v_{1}$ and $v_{2}$ for an exemplary NOMA scheme for $R_{1}=1$ bps/Hz, $R_{2}=1$ bps/Hz, $\lambda_{m}=0.75$, and $v_{2,\mathrm{th}}=0.4$, where the quantities $v_{1}^{c}$, $v_{1}^{\mathrm{im}}$, $v_{1,\mathrm{th}}^{p}$, $v_{1,\mathrm{th}}^{m}$, $v_{1}^{\mathrm{pm}}$, $v_{1}^{\mathrm{zm}}$, and $v_{1}^{\mathrm{zp}}$ are the values of $v_{1}$ at the intersection points. See Table 1 for detailed explanations of these quantities.

Next, the following theorem provides a detailed characterization of the outage probability of the SU, $P_{\mathrm{out},1}$, which helps to find the optimal $v_{1}$ and $v_{2}$ to minimize it.

**Theorem** **1.** *Given that $v_{2}$ is a monotonically decreasing function of $v_{1}$, and $\left(-\frac{dv_{2}}{dv_{1}}\right)$ is a non-decreasing function of $v_{1}$. Let $v_{1}^{d}=\arg\min_{v_{1}}P_{\mathrm{out},1}$. Then $P_{\mathrm{out},1}$ monotonically decreases in $v_{1}$ when $v_{1}\leq v_{1}^{d}$ and increases when $v_{1}\geq v_{1}^{d}$, and $v_{1}^{d}>v_{e}>v_{2}\left(v_{1}^{d}\right)$, where $v_{e}$ is the value of $v_{1}$, such that $v_{2}=v_{1}$.*

**Proof.**  The derivative of $P_{\mathrm{out},1}$ in (28) with respect to $v_{1}$ is given by

(47) $$\begin{array}{cc} \frac{dP_{\mathrm{out},1}}{dv_{1}}=\; & \frac{1}{p_{2}{\bar{\gamma }}_{1}v_{2}}\exp\left[-\frac{1}{{\bar{\gamma }}_{1}p_{2}\min\left(v_{1},v_{2}\right)}-\frac{1}{p_{2}\xi {\bar{\gamma }}_{1}}\max\left(\frac{v_{1}}{v_{2}}-1,0\right)\right]\\ \; & \times \underset{D\left(v_{1}\right)}{\underset{︸}{\left\{\frac{1}{v_{2}}\left[\exp\left[\frac{1}{p_{2}\xi {\bar{\gamma }}_{1}}\max\left(\frac{v_{1}}{v_{2}}-1,0\right)\right]-1\right]\left(-\frac{dv_{2}}{dv_{1}}\right)-\frac{\left(v_{1}+\xi \right)+p_{2}{\bar{\gamma }}_{1}\xi v_{2}}{{\left(v_{1}+\xi \right)}^{2}}\right\}}}, \end{array}$$

where $D(v_{1})$ is an increasing function of $v_{1}$ that is smaller than zero when $v_{1}\leq v_{e}$ and goes to infinity when $v_{1}/v_{2}\to \infty$. Therefore, there is a unique $v_{1}^{d}$ that makes $D(v_{1}^{d})=0$, and $v_{1}^{d}>v_{e}$. When $v_{1}<v_{1}^{d}$, we have $\frac{dP_{\mathrm{out},1}}{dv_{1}}<0$; thus, $P_{\mathrm{out},1}$ decreases in $v_{1}$. When $v_{1}>v_{1}^{d}$, we have $\frac{dP_{\mathrm{out},1}}{dv_{1}}>0$ and, thus, $P_{\mathrm{out},1}$ increases in $v_{1}$. □

### 4.1. PGS Design

In the PGS design, the impropriety degree of the SU κ≡0 and, thus, $v_{2}\equiv v_{2}^{p}\left(v_{1}\right)$, where $v_{1}$ has to meet the constraints in (43). Note that the first condition in (43), i.e., $v_{2}^{L}\left(v_{1}\right)\leq v_{2}\leq v_{2}^{U}\left(v_{1}\right)$, holds as long as $0<v_{1}\leq v_{1}^{\mathrm{pm}}$, so this condition is irrelevant to the range of $v_{1}$. Therefore, the original problem can be equivalently expressed as

(48) $$\begin{array}{cc} \min_{v_{1}}\; & P_{\mathrm{out},1}\\ \mathrm{subject}\;\mathrm{to}\; & v_{2}^{p}\left(v_{1}\right)\geq v_{2,\mathrm{th}},\\ \; & 0<v_{1}\leq v_{1}^{\mathrm{pm}}. \end{array}$$

According to Theorem 1, since

(49) $$-\frac{dv_{2}^{p}\left(v_{1}\right)}{dv_{1}}=\sqrt{1+\Gamma_{1}}-1$$

is a constant, there exists a $v_{1}^{\mathrm{dp}}$, such that $P_{\mathrm{out},1}$ decreases in $v_{1}$ when $0<v_{1}\leq v_{1}^{\mathrm{dp}}$ and increases in $v_{1}$ when $v_{1}\geq v_{1}^{\mathrm{dp}}$, and $v_{1}^{\mathrm{dp}}>v_{e}^{p}$, where

(50) $$v_{e}^{p}=\frac{1}{\sqrt{1+\Gamma_{1}}\left(\sqrt{1+\Gamma_{2}}-1\right)}$$

is the value of $v_{1}$, making $v_{2}^{p}\left(v_{1}\right)=v_{1}$. The substitution of $v_{2}^{p}\left(\cdot \right)$ in (36) into the first constraint in (48) yields

(51) $$v_{1}\leq v_{1,\mathrm{th}}^{p}≜\frac{1}{\sqrt{1+\Gamma_{1}}-1}\left(\frac{1}{\sqrt{1+\Gamma_{2}}-1}-v_{2,\mathrm{th}}\right).$$

Therefore, the optimal $v_{1}=\min\left\{v_{1}^{\mathrm{pm}},v_{1,\mathrm{th}}^{p},v_{1}^{\mathrm{dp}}\right\}$. Note that by replacing $-\frac{dv_{2}}{dv_{1}}$ in D(·) given in (47) with the expression in (49), we obtain

(52) $$\begin{array}{cc} D^{p}\left(v_{1}\right)≜\; & \frac{\sqrt{1+\Gamma_{1}}-1}{v_{2}^{p}\left(v_{1}\right)}\left[\exp\left[\frac{1}{p_{2}\xi {\bar{\gamma }}_{1}}\max\left(\frac{v_{1}}{v_{2}^{p}\left(v_{1}\right)}-1,0\right)\right]-1\right]\\ \; & -\frac{\left(v_{1}+\xi \right)+p_{2}{\bar{\gamma }}_{1}\xi v_{2}^{p}\left(v_{1}\right)}{{\left(v_{1}+\xi \right)}^{2}}, \end{array}$$

which is a monotonically increasing function, and $D^{p}\left(v_{1}^{\mathrm{dp}}\right)=0$, so that the optimal $v_{1}$ is given by

(53) $$v_{1}^{\mathrm{op}}=\left\{\begin{array}{cc} \min\left\{v_{1}^{\mathrm{pm}},v_{1,\mathrm{th}}^{p}\right\}, & D^{p}\left(\min\left\{v_{1}^{\mathrm{pm}},v_{1,\mathrm{th}}^{p}\right\}\right)\leq 0,\\ v_{1}^{\mathrm{dp}}, & D^{p}\left(\min\left\{v_{1}^{\mathrm{pm}},v_{1,\mathrm{th}}^{p}\right\}\right)>0, \end{array}\right.$$

where $v_{1}^{\mathrm{dp}}$ can be obtained with a bisection search, and the optimal transmit power of the SU for minimizing $P_{\mathrm{out},1}$ in the PGS design is given by $p_{1}^{\mathrm{op}}=v_{1}^{\mathrm{op}}p_{2}\left(\sqrt{1+\Gamma_{1}}-1\right)$.

### 4.2. IGS Design

When the SU adopts IGS, the quantities $v_{1}$ and $v_{2}$ are jointly optimized to minimize $P_{\mathrm{out},1}$ subject to the constraints given in (43). To solve the optimization problem (29) with two variables, we first represent one of the variables with the other one, so as to convert the original problem to a single-variable optimization. Since both $P_{\mathrm{out},1}$ and $P_{\mathrm{out},2}$ decrease in $v_{2}$, we first set $v_{2}=v_{2}^{U}\left(v_{1}\right)$. Similar to the analysis in Section 4.1, the first condition in (43), i.e., $v_{2}^{L}\left(v_{1}\right)\leq v_{2}\leq v_{2}^{U}\left(v_{1}\right)$, is irrelevant to the range of $v_{1}$. Therefore, the original problem (29) can be simplified to

(54) $$\begin{array}{cc} \min_{v_{1}}\; & P_{\mathrm{out},1}\\ \mathrm{subject}\;\mathrm{to}\; & v_{2}^{U}\left(v_{1}\right)\geq v_{2,\mathrm{th}},\;0<v_{1}\leq v_{1}^{\mathrm{pm}}. \end{array}$$

Since all three functions $v_{2}^{p}\left(\cdot \right)$, $v_{2}^{i}\left(\cdot \right)$, and $v_{2}^{m}\left(\cdot \right)$ defined in (36) are decreasing functions, $v_{2}^{U}\left(\cdot \right)$ given in (45) is also decreasing. Therefore, the conditions in (54) can be equivalently written as $0<v_{1}\leq v_{1,\mathrm{th}}$, where

(55) $$v_{1,\mathrm{th}}≜\left\{\begin{array}{cc} v_{1,\mathrm{th}}^{p}, & v_{2,\mathrm{th}}\geq \max\left\{v_{2}^{c},v_{2}^{p}\left(v_{1}^{\mathrm{pm}}\right)\right\},\\ v_{1,\mathrm{th}}^{i}, & v_{2}^{i}\left(v_{1}^{\mathrm{im}}\right)<v_{2,\mathrm{th}}<v_{2}^{c},\\ v_{1,\mathrm{th}}^{m}, & v_{2}^{p}\left(v_{1}^{\mathrm{pm}}\right)\leq v_{2,\mathrm{th}}\leq \min\left\{v_{2}^{i}\left(v_{1}^{\mathrm{im}}\right),v_{2}^{c}\right\},\\ v_{1}^{\mathrm{pm}}, & v_{2,\mathrm{th}}<v_{2}^{p}\left(v_{1}^{\mathrm{pm}}\right), \end{array}\right.$$

$v_{1,\mathrm{th}}^{i}$ and $v_{1,\mathrm{th}}^{m}$ are the solutions to $v_{2}^{i}\left(\cdot \right)=v_{2,\mathrm{th}}$ and $v_{2}^{m}\left(\cdot \right)=v_{2,\mathrm{th}}$, given by

(56) $$v_{1,\mathrm{th}}^{i}≜\frac{1+\Gamma_{2}-{\left(\Gamma_{2}v_{2,\mathrm{th}}-1\right)}^{2}}{\Gamma_{1}\Gamma_{2}\left(\Gamma_{2}v_{2,\mathrm{th}}-1\right)}$$

and

(57) $$v_{1,\mathrm{th}}^{m}≜\frac{\lambda_{m}+\sqrt{\lambda_{m}^{2}-\Gamma_{1}\left[{\left(v_{2,\mathrm{th}}-\frac{1}{\Gamma_{2}}+\lambda_{m}\right)}^{2}-\lambda_{m}^{2}-\frac{1+\Gamma_{2}}{\Gamma_{2}^{2}}\right]}}{\Gamma_{1}},$$

respectively.

Since $v_{2}^{U}\left(v_{1}\right)$ given in (45) can be any of $v_{2}^{p}\left(v_{1}\right)$, $v_{2}^{i}\left(v_{1}\right)$, or $v_{2}^{m}\left(v_{1}\right)$, three cases are studied in the sequel to determine the optimal $v_{1}$:

1. $v_{2}^{U}\left(v_{1}\right)=v_{2}^{p}\left(v_{1}\right)$.
  In this case, we have $v_{1}\leq v_{1}^{c}$, and the IGS scheme reduces to the PGS scheme introduced in Section 4.1. Since $v_{1}^{c}<v_{2}^{c}=v_{2}^{p}\left(v_{1}^{c}\right)$, $v_{e}^{p}=v_{2}^{p}\left(v_{e}^{p}\right)$, and $v_{2}^{p}\left(\cdot \right)$ is a decreasing function, we have $v_{1}^{c}<v_{e}^{p}<v_{1}^{\mathrm{dp}}$; thus, $P_{\mathrm{out},1}$ decreases in $v_{1}$ as $v_{1}\leq v_{1}^{c}$. If $v_{1,\mathrm{th}}\leq v_{1}^{c}$, then the optimal solution to (54) is given by $v_{1}^{★}=v_{1,\mathrm{th}}$, and the optimal power and impropriety degree of the SU are $p_{1}^{★}=p_{1}^{\mathrm{op}}=v_{1,\mathrm{th}}p_{2}\left(\sqrt{1+\Gamma_{1}}-1\right)$ and $\kappa^{★}=0$, respectively, which are the same as the results presented in Section 4.1. Otherwise, if $v_{1,\mathrm{th}}>v_{1}^{c}$, then the optimal $v_{1}$ that minimizes the SU outage probability $P_{\mathrm{out},1}$ cannot be achieved in $\left(0,v_{1}^{c}\right]$.
2. $v_{2}^{U}\left(v_{1}\right)=v_{2}^{m}\left(v_{1}\right)$.
  In this case, we have $v_{1}\geq v_{1}^{c}$ and $v_{2}^{m}\left(v_{1}\right)\leq v_{2}^{i}\left(v_{1}\right)$, i.e., $\max\left(v_{1}^{c},v_{1}^{\mathrm{im}}\right)\leq v_{1}\leq v_{1,\mathrm{th}}$. Since
  (58) $$\frac{d}{dv_{1}}\left(-\frac{dv_{2}^{m}\left(v_{1}\right)}{dv_{1}}\right)=\frac{\left(\Gamma_{1}+1\right)\lambda_{m}^{2}+\Gamma_{1}\frac{1+\Gamma_{2}}{\Gamma_{2}^{2}}}{{\left(\lambda_{m}^{2}+2\lambda_{m}v_{1}-\Gamma_{1}v_{1}^{2}+\frac{1+\Gamma_{2}}{\Gamma_{2}^{2}}\right)}^{3/2}}>0,$$
  there exists a $v_{1}^{\mathrm{dm}}$, such that $P_{\mathrm{out},1}$ decreases in $v_{1}$ when $v_{1}\leq v_{1}^{\mathrm{dm}}$ and increases when $v_{1}\geq v_{1}^{\mathrm{dm}}$ according to Theorem 1. Moreover, we have $v_{1}^{\mathrm{dm}}\geq v_{2}^{m}\left(v_{1}^{\mathrm{dm}}\right)$ and $D^{m}\left(v_{1}^{\mathrm{dm}}\right)=0$, where
  (59) $$\begin{array}{cc} D^{m}\left(v_{1}\right)≜\; & \frac{1}{v_{2}^{m}\left(v_{1}\right)}\left[\exp\left[\frac{1}{p_{2}\xi {\bar{\gamma }}_{1}}\max\left(\frac{v_{1}}{v_{2}^{m}\left(v_{1}\right)}-1,0\right)\right]-1\right]\\ \; & \times \left(-\frac{dv_{2}^{m}\left(v_{1}\right)}{dv_{1}}\right)-\frac{\left(v_{1}+\xi \right)+p_{2}{\bar{\gamma }}_{1}\xi v_{2}^{m}\left(v_{1}\right)}{{\left(v_{1}+\xi \right)}^{2}}, \end{array}$$
  (60) $$-\frac{dv_{2}^{m}\left(v_{1}\right)}{dv_{1}}=\frac{\Gamma_{1}v_{1}-\lambda_{m}}{\sqrt{\lambda_{m}^{2}+2\lambda_{m}v_{1}-\Gamma_{1}v_{1}^{2}+\frac{1+\Gamma_{2}}{\Gamma_{2}^{2}}}}.$$
  Therefore, if $\max\left(v_{1}^{c},v_{1}^{\mathrm{im}}\right)\leq v_{1,\mathrm{th}}$, then the optimal $v_{1}$ in $\left[\max\left(v_{1}^{c},v_{1}^{\mathrm{im}}\right),v_{1,\mathrm{th}}\right]$ that minimizes $P_{\mathrm{out},1}$ can be expressed as
  (61) $$v_{1}^{\mathrm{om}}=\left\{\begin{array}{cc} v_{1}^{\mathrm{im}}, & D^{m}\left(v_{1}^{\mathrm{im}}\right)\geq 0,\\ v_{1,\mathrm{th}}, & D^{m}\left(v_{1,\mathrm{th}}\right)\leq 0,\\ v_{1}^{\mathrm{dm}}, & \mathrm{otherwise}, \end{array}\right.$$
  where $v_{1}^{\mathrm{dm}}$ can be obtained with the bisection search.
3. $v_{2}^{U}\left(v_{1}\right)=v_{2}^{i}\left(v_{1}\right)$.
  In this case, we have $v_{1}^{c}\leq v_{1}\leq \min\left(v_{1}^{\mathrm{im}},v_{1,\mathrm{th}}\right)$. Unfortunately, the optimization is not simple compared with the previous cases, which have some interesting monotonic characteristics. However, inspired by the solution trend of the previous cases, $v_{1}$ can be given by
  (62) $$v_{1}^{\mathrm{oi}}=\left\{\begin{array}{cc} \min\left(v_{1}^{\mathrm{im}},v_{1,\mathrm{th}}\right), & D^{i}\left(\min\left(v_{1}^{\mathrm{im}},v_{1,\mathrm{th}}\right)\right)\leq 0,\\ v_{1}^{\mathrm{di}}, & D^{i}\left(\min\left(v_{1}^{\mathrm{im}},v_{1,\mathrm{th}}\right)\right)>0, \end{array}\right.$$
  where $v_{1}^{\mathrm{di}}$ is the solution to $D^{i}\left(v_{1}\right)=0$ with $D^{i}\left(\cdot \right)$ given by
  (63) $$\begin{array}{cc} D^{i}\left(v_{1}\right)≜\; & \frac{1}{v_{2}^{i}\;\left(v_{1}\right)}\;\left[\exp\;\left[\frac{1}{p_{2}\xi {\bar{\gamma }}_{1}}\max\;\left(\frac{v_{1}}{v_{2}^{i}\;\left(v_{1}\right)}\;-\;1,0\right)\right]-1\right]\\ \; & \times \left(-\frac{dv_{2}^{i}\left(v_{1}\right)}{dv_{1}}\right)-\frac{\left(v_{1}+\xi \right)+p_{2}{\bar{\gamma }}_{1}\xi v_{2}^{i}\left(v_{1}\right)}{{\left(v_{1}+\xi \right)}^{2}}, \end{array}$$
  in which
  (64) $$-\frac{dv_{2}^{i}\left(v_{1}\right)}{dv_{1}}=\frac{\Gamma_{1}}{2}-\frac{\Gamma_{1}^{2}v_{1}}{2\sqrt{4\frac{1+\Gamma_{2}}{\Gamma_{2}^{2}}+\Gamma_{1}^{2}v_{1}^{2}}}.$$
  Likewise, $v_{1}^{\mathrm{di}}$ can be obtained with the bisection search.

In summary, the solution to (54) can be given by

(65) $$v_{1}^{★}=\left\{\begin{array}{cc} v_{1,\mathrm{th}}, & v_{1,\mathrm{th}}\leq v_{1}^{c},\\ v_{1}^{\mathrm{om}}, & v_{1}^{\mathrm{im}}\leq v_{1}^{c}<v_{1,\mathrm{th}},\\ v_{1}^{\mathrm{oi}}, & v_{1}^{c}<v_{1,\mathrm{th}}\leq v_{1}^{\mathrm{im}},\\ \arg\min_{v_{1}\in \left\{v_{1}^{\mathrm{om}},v_{1}^{\mathrm{oi}}\right\}}P_{\mathrm{out},1}, & v_{1}^{c}<v_{1}^{\mathrm{im}}<v_{1,\mathrm{th}}. \end{array}\right.$$

Consider the situation that $v_{1}^{c}<v_{1}^{\mathrm{im}}<v_{1,\mathrm{th}}$. Since $P_{\mathrm{out},1}\left(v_{1},v_{2}\right)$ is non-increasing in $v_{2}$, when $v_{1}>v_{1}^{\mathrm{im}}$, we have $v_{2}^{i}\left(v_{1}\right)>v_{2}^{m}\left(v_{1}\right)$ and $P_{\mathrm{out},1}\left(v_{1},v_{2}^{i}\left(v_{1}\right)\right)\leq P_{\mathrm{out},1}\left(v_{1},v_{2}^{m}\left(v_{1}\right)\right)$, so when $v_{1}=v_{1}^{\mathrm{im}}$, we have $\frac{d}{dv_{1}}P_{\mathrm{out},1}\left(v_{1},v_{2}^{i}\left(v_{1}\right)\right)\leq \frac{d}{dv_{1}}P_{\mathrm{out},1}\left(v_{1},v_{2}^{m}\left(v_{1}\right)\right)$ and, thus, $D^{i}\left(v_{1}^{\mathrm{im}}\right)\leq D^{m}\left(v_{1}^{\mathrm{im}}\right)$ according to (47), (59), and (63). By combining (61) and (62), the optimal $v_{1}$ can be given by

(66) $$\arg\min_{v_{1}\in \left\{v_{1}^{\mathrm{om}},v_{1}^{\mathrm{oi}}\right\}}P_{\mathrm{out},1}=\left\{\begin{array}{cc} v_{1}^{\mathrm{di}}, & D^{i}\left(v_{1}^{\mathrm{im}}\right)>0,\\ v_{1}^{\mathrm{im}}, & D^{i}\left(v_{1}^{\mathrm{im}}\right)\leq 0\leq D^{m}\left(v_{1}^{\mathrm{im}}\right),\\ v_{1,\mathrm{th}}, & D^{m}\left(v_{1,\mathrm{th}}\right)\leq 0,\\ v_{1}^{\mathrm{dm}}, & \mathrm{otherwise}. \end{array}\right.$$

By combining (65) and (66), the solution to (54) can be expressed as

(67) $$v_{1}^{★}=\left\{\begin{array}{cc} v_{1,\mathrm{th}}, & v_{1}^{\mathrm{im}}\leq v_{1,\mathrm{th}}\;\mathrm{and}\;D^{m}\left(v_{1,\mathrm{th}}\right)\leq 0,\;\mathrm{or},\;v_{1}^{\mathrm{im}}>v_{1,\mathrm{th}}\;\mathrm{and}\;D^{i}\left(v_{1,\mathrm{th}}\right)\leq 0,\\ v_{1}^{\mathrm{im}}, & v_{1}^{\mathrm{im}}\leq v_{1,\mathrm{th}}\;\mathrm{and}\;D^{i}\left(v_{1}^{\mathrm{im}}\right)\leq 0\leq D^{m}\left(v_{1}^{\mathrm{im}}\right),\\ v_{1}^{\mathrm{di}}, & D^{i}\left(\min\left(v_{1}^{\mathrm{im}},v_{1,\mathrm{th}}\right)\right)>0,\\ v_{1}^{\mathrm{dm}}, & \mathrm{otherwise}, \end{array}\right.$$

where both $v_{1}^{\mathrm{di}}\in \left(v_{1}^{c},\min\left(v_{1}^{\mathrm{im}},v_{1,\mathrm{th}}\right)\right]$ and $v_{1}^{\mathrm{dm}}\in \left[\max\left(v_{1}^{\mathrm{im}},v_{1}^{c}\right),v_{1,\mathrm{th}}\right]$ can be obtained with the bisection search. The corresponding power and impropriety degree of the SU, i.e., $p_{1}$ and κ, of the proposed IGS design for minimizing the outage probability of the SU, $P_{\mathrm{out},1}$, are given by

(68) $$p_{1}^{★}=\left\{\begin{array}{cc} v_{1}^{★}p_{2}\left(\sqrt{1+\Gamma_{1}}-1\right), & v_{1}^{★}\leq v_{1}^{c},\\ v_{1}^{★}p_{2}\Gamma_{1}/2, & v_{1}^{c}<v_{1}^{★}\leq v_{1}^{\mathrm{im}},\\ p_{1,\max}, & v_{1}^{★}>\max\left(v_{1}^{c},v_{1}^{\mathrm{im}}\right) \end{array}\right.$$

and

(69) $$\kappa^{★}=\left\{\begin{array}{cc} 0, & v_{1}^{★}\leq v_{1}^{c},\\ 1, & v_{1}^{c}<v_{1}^{★}\leq v_{1}^{\mathrm{im}},\\ \sqrt{1-\frac{v_{1}^{★}p_{2}}{p_{1,\max}}\left(\frac{\Gamma_{1}v_{1}^{★}p_{2}}{p_{1,\max}}-2\right)}, & v_{1}^{★}>\max\left(v_{1}^{c},v_{1}^{\mathrm{im}}\right), \end{array}\right.$$

respectively.

Based on the above analysis, the IGS design outperforms the PGS design if $v_{2}^{U}\left(v_{1}^{★}\right)\neq v_{2}^{p}\left(v_{1}^{★}\right)$, or equivalently, $v_{1}^{c}<v_{1}^{★}<v_{1}^{\mathrm{pm}}$ according to (45). Since $v_{1}^{★}$ is given in (67), this condition can also be expressed as, $v_{1,\mathrm{th}}>v_{1}^{c}$, and either $v_{1,\mathrm{th}}<v_{1}^{\mathrm{pm}}$ or $D^{m}\left(v_{1}^{\mathrm{pm}}\right)>0$ is satisfied. According to (55), if $v_{2,\mathrm{th}}<v_{2}^{c}$ and either $v_{2,\mathrm{th}}>v_{2}^{p}\left(v_{1}^{\mathrm{pm}}\right)$ or $D^{m}\left(v_{1}^{\mathrm{pm}}\right)>0$ is guaranteed, then the IGS design can achieve a lower outage probability of the SU than the PGS design.

### 4.3. Optimal Outage Performance in Extreme SNR

In what follows, the power ratio $\lambda_{m}=p_{1,\max}/p_{2}$ is assumed to be a constant in order to investigate how the SNR affects the optimality of our proposed IGS scheme. Note that all the expressions of $v_{2}^{p}\left(\cdot \right)$, $v_{2}^{i}\left(\cdot \right)$, and $v_{2}^{m}\left(\cdot \right)$ given in (36) are irrelevant to the value of $p_{2}$, so $v_{1}^{c}$, $v_{1}^{\mathrm{pm}}$, and $v_{1}^{\mathrm{im}}$ all remain constant as $p_{2}$ increases. However, both $v_{2,\mathrm{th}}$ defined in (31) and $D(v_{1})$ given in (47) monotonically decrease in $p_{2}$.

At low SNRs, $v_{2,\mathrm{th}}$ reaches a greater value. The PGS design achieves the optimal outage performance of the SU when the SNR is low enough so that $v_{2,\mathrm{th}}\geq v_{2}^{c}$, or equivalently, $p_{2}\leq -{\left[{\bar{\gamma }}_{2}v_{2}^{c}\log\left(1-P_{\mathrm{out},\mathrm{th}}\right)\right]}^{-1}$. Therefore, the IGS design is unlikely to provide better outage performance than the traditional PGS design at a low SNR.

In contrast, at high SNRs, $v_{2,\mathrm{th}}$ reaches a smaller value and even approaches zero as $p_{2}\to \infty$. According to (55), $v_{1,\mathrm{th}}$ can be expressed as

(70) $$\begin{array}{cc} v_{1,\mathrm{th}}=\; & \left\{\begin{array}{cc} v_{1,\mathrm{th}}^{m}, & v_{2,\mathrm{th}}\geq v_{2}^{p}\left(v_{1}^{\mathrm{pm}}\right),\\ v_{1}^{\mathrm{pm}}, & v_{2,\mathrm{th}}<v_{2}^{p}\left(v_{1}^{\mathrm{pm}}\right) \end{array}\right.\\ =\; & \min\left\{v_{1,\mathrm{th}}^{m},v_{1}^{\mathrm{pm}}\right\} \end{array}$$

at a high SNR, which is greater than $v_{1}^{\mathrm{im}}$ since $v_{2}^{m}\left(\cdot \right)$ is decreasing and $v_{2,\mathrm{th}}=v_{2}^{m}\left(v_{1,\mathrm{th}}^{m}\right)<v_{2}^{m}\left(v_{1}^{\mathrm{im}}\right)$. when $p_{2}$ is large enough so that both $D^{i}\left(v_{1}^{\mathrm{im}}\right)$ and $D^{i}\left(v_{1,\mathrm{th}}\right)$ are negative, according to (67), $v_{1}^{★}$ can be given by

(71) $$v_{1}^{★}=\left\{\begin{array}{cc} v_{1,\mathrm{th}}, & D^{m}\left(v_{1,\mathrm{th}}\right)\leq 0,\\ v_{1}^{\mathrm{dm}}, & \mathrm{otherwise}. \end{array}\right.$$

Note that (71) has a similar form to the expression of $v_{1}^{\mathrm{op}}$ given in (53). According to (28), as $p_{2}$ approaches infinity, the outage probability of the SU converges to

(72) $$\lim_{p_{2}\to \infty }P_{\mathrm{out},1}=\frac{\xi }{v_{1}+\xi }.$$

If $v_{2}^{p}\left(v_{1}^{\mathrm{pm}}\right)\leq 0$, i.e., $\lambda_{m}\geq {\left(\sqrt{1+\Gamma_{2}}-1\right)}^{-1}$ according to (36) and (39), then $v_{2,\mathrm{th}}>0\geq v_{2}^{p}\left(v_{1}^{\mathrm{pm}}\right)$, so the IGS design would continue to achieve a lower outage probability of the SU than the PGS design as $p_{2}$ approaches infinity.

In the PGS design, since $v_{2}^{p}\left(\cdot \right)$ is a decreasing function, and $v_{2,\mathrm{th}}=v_{2}^{p}\left(v_{1,\mathrm{th}}^{p}\right)>v_{2}^{p}\left(v_{1}^{\mathrm{pm}}\right)$, we have $v_{1}^{\mathrm{pm}}>v_{1,\mathrm{th}}^{p}$ and, thus, $\min\left\{v_{1}^{\mathrm{pm}},v_{1,\mathrm{th}}^{p}\right\}=v_{1,\mathrm{th}}^{p}\to v_{1}^{\mathrm{zp}}$ as $p_{2}\to \infty$, where $v_{1}^{\mathrm{zp}}$ is the solution to $v_{2}^{p}\left(\cdot \right)=0$, given by

(73) $$v_{1}^{\mathrm{zp}}=\frac{1}{\left(\sqrt{1+\Gamma_{1}}-1\right)\left(\sqrt{1+\Gamma_{2}}-1\right)}.$$

Since $D^{p}\left(v_{1}\right)$ approaches −∞ as $p_{2}\to \infty$ and approaches *∞* as $v_{1}\to v_{1}^{\mathrm{zp}}$, the solution to $D^{p}\left(\cdot \right)=0$, i.e., $v_{1}^{\mathrm{dp}}$, approaches $v_{1}^{\mathrm{zp}}$ as $p_{2}\to \infty$. Therefore, $v_{1}^{\mathrm{op}}$ given in (53) approaches $v_{1}^{\mathrm{zp}}$ and, consequently, the outage probability of the SU, $P_{\mathrm{out},1}$, approaches $\xi /(v_{1}^{\mathrm{zp}}+\xi )$ as $p_{2}\to \infty$. In the IGS design, according to (70), the quantity $v_{1,\mathrm{th}}=v_{1,\mathrm{th}}^{m}\to v_{1}^{\mathrm{zm}}$ as $p_{2}\to \infty$, where

(74) $$v_{1}^{\mathrm{zm}}=\frac{\lambda_{m}+\sqrt{\lambda_{m}^{2}+\Gamma_{1}\left(2\lambda_{m}+1\right)/\Gamma_{2}}}{\Gamma_{1}}$$

is the solution to $v_{2}^{m}\left(\cdot \right)=0$. Similarly, since $D^{m}\left(v_{1}\right)$ approaches −∞ as $p_{2}\to \infty$ and approaches *∞* as $v_{1}\to v_{1}^{\mathrm{zm}}$, according to (71), the quantity $v_{1}^{★}\to v_{1}^{\mathrm{zm}}$ and, thus, $P_{\mathrm{out},1}$ approaches $\xi /(v_{1}^{\mathrm{zm}}+\xi )$ as $p_{2}\to \infty$. When $v_{2}^{p}\left(v_{1}^{\mathrm{pm}}\right)<0$, we have $v_{1}^{\mathrm{zm}}>v_{1}^{\mathrm{zp}}$; therefore, the IGS design always yields an outage probability of the SU that is strictly smaller than that of the PGS design. This indicates that the IGS design outperforms the PGS design at a high SNR with a high target rate of the WU or a high maximum allowable power ratio between the SU and the WU.

Otherwise, if $v_{2}^{p}\left(v_{1}^{\mathrm{pm}}\right)>0$, i.e., $\lambda_{m}<{\left(\sqrt{1+\Gamma_{2}}-1\right)}^{-1}$, then a sufficiently high SNR would make $v_{2,\mathrm{th}}<v_{2}^{p}\left(v_{1}^{\mathrm{pm}}\right)$, so $v_{1,\mathrm{th}}=v_{1}^{\mathrm{pm}}$ according to (70). Since $D^{m}\left(v_{1}^{\mathrm{pm}}\right)\to -\infty$ as $p_{2}\to \infty$, the optimal $v_{1}$ is $v_{1}^{★}=v_{1}^{\mathrm{pm}}$, and, thus, the optimal outage performance of the SU is achieved by the PGS with the maximum power budget of the SU, and the IGS design reduces to the PGS one with $P_{\mathrm{out},1}$ approaching $\xi /(v_{1}^{\mathrm{pm}}+\xi )$ as the SNR increases. Therefore, the proposed IGS design outperforms the PGS design only in a certain SNR range.

## 5. Numerical Results

In order to evaluate the benefits of our designed IGS scheme for the downlink two-user NOMA system under imperfect SIC, numerical experiments were conducted in the MATLAB programming environment. Throughout the comparisons between PGS and IGS schemes, the effects of different system parameters on the benefits of the IGS-based system over the PGS-based system were highlighted. The experiments simulated both users’ outage probabilities for both the PGS- and IGS-based systems versus the SNR of the WU, assuming the different maximum power ratios between two users $\lambda_{m}$, levels of residual interference due to imperfect SIC ξ, and CNR ratios between two users ${\bar{\gamma }}_{1}/{\bar{\gamma }}_{2}$. The SNR of the WU was defined as $p_{2}/\sigma_{n_{2}}^{2}$. Unless otherwise specified, the simulation parameters were set according to Table 2. In the PGS scheme, both $v_{1}^{\mathrm{op}}$ and $v_{2}^{p}\left(v_{1}^{\mathrm{op}}\right)$ were calculated according to (53) and (36) to obtain both users’ outage probabilities, given in (21) and (28). In the IGS scheme, we calculated $v_{1}^{★}$ according to (67) and $v_{2}^{U}\left(v_{1}^{★}\right)$ according to (45), and obtained both users’ outage probabilities accordingly.

**Figure 3:**
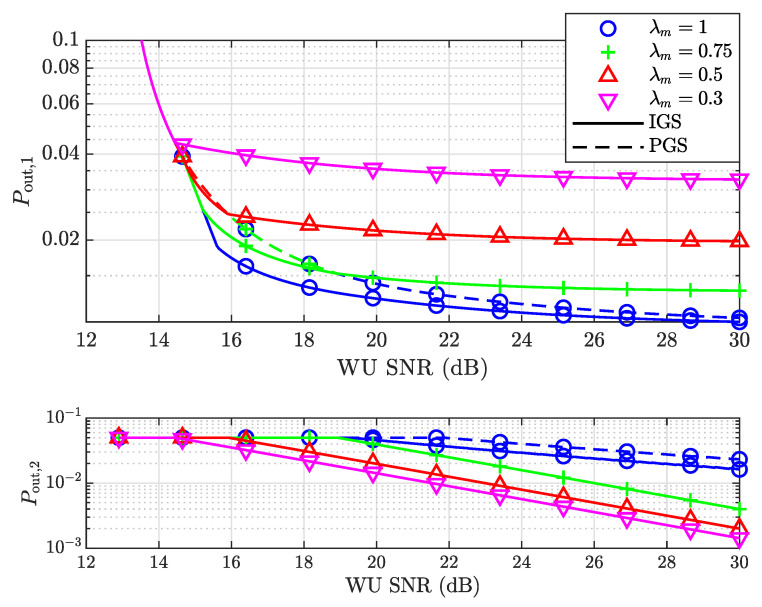
The outage probabilities of both the strong user (SU) and weak user (WU) versus the signal-to-noise ratio (SNR) of the WU for different $\lambda_{m}$ when $R_{1}=1$ bps/Hz, $R_{2}=1$ bps/Hz.

We first simulated the outage probabilities of both users for both the PGS- and IGS-based systems, assuming different maximum power ratios $\lambda_{m}$, given $R_{1}=R_{2}=1$ bps/Hz, as shown in Figure 3. The outage performance of the SU in both the PGS and IGS schemes improves with the increase in SNR. When the SNR of the WU is lower than 14.4 dB, both the PGS and IGS schemes at different $\lambda_{m}$ share the same outage probabilities of the WU. This is because $p_{2}$ is small enough, so that $v_{2,\mathrm{th}}$ is no smaller than both $v_{2}^{c}$ and $v_{2}^{p}\left(v_{1}^{\mathrm{pm}}\right)$, making $v_{1}^{★}=v_{1}^{\mathrm{op}}\leq v_{1,\mathrm{th}}<v_{1}^{\mathrm{pm}}$. Thus, the PGS scheme achieves the optimal outage performance with $p_{1}<p_{1,\max}=\lambda_{m}p_{2}$, indicating that the minimum achievable $P_{\mathrm{out},1}$ is irrelevant to $\lambda_{m}$. As the SNR increases, the outage probability of the SU under the PGS scheme continues to decrease and eventually saturates at high SNR when $p_{1}$ reaches $p_{1,\max}$. In comparison, the IGS scheme achieves a lower outage probability of the SU for $\lambda_{m}\geq 0.75$, but the benefit of using IGS eventually decreases and even disappears as the SNR increases, i.e., the IGS scheme outperforms PGS only in a certain range of SNR, which is consistent with the analysis given at the end of Section 4.3. It can also be observed that the IGS scheme is more likely to outperform the PGS one with a larger $\lambda_{m}=p_{1,\max}/p_{2}$, i.e., a higher maximum power threshold of the SU. This result is consistent with the one in [36], in that improper signaling can achieve higher fairness with a higher power allocation factor of the SU. The outage probability of the WU is equal to its maximum threshold $P_{\mathrm{out},\mathrm{th}}$ at a low SNR. As the SNR increases, the WU achieves a lower outage probability while satisfying the optimal outage performance of the SU. When $\lambda_{m}<1$, since $v_{2}^{p}\left(v_{1}^{\mathrm{pm}}\right)>0$, according to Section 4.3, the optimal outage performance of the SU is achieved by the PGS design with the maximum power of the SU, $p_{1,\max}$, and $P_{\mathrm{out},2}$ is inversely proportional to SNR and identical in both the PGS and IGS designs. However, when $\lambda_{m}=1$, we have $v_{2}^{p}\left(v_{1}^{\mathrm{pm}}\right)=0$; therefore, $P_{\mathrm{out},2}$ decreases more slowly. The IGS-based system achieves a lower outage probability of the WU.

**Figure 4:**
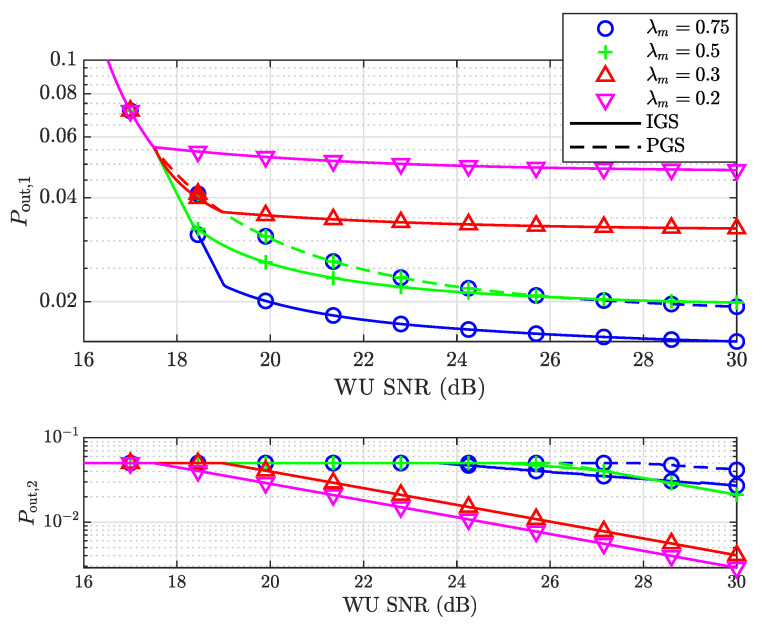
The outage probabilities of both the SU and WU versus the SNR of the WU for different $\lambda_{m}$ when $R_{1}=1$ bps/Hz, $R_{2}=1.5$ bps/Hz.

Next, we considered another pair of the target rates of both users, given by $R_{1}=1$ bps/Hz and $R_{2}=1.5$ bps/Hz. The outage probabilities of both users versus the SNR of the WU are shown in Figure 4. It is clear that for $\lambda_{m}=0.75$, the outage probabilities of the SU under PGS and IGS saturate at different values at high SNR. The IGS achieves a lower outage probability, and the gap between the IGS and PGS probabilities is about 0.005. This is because $v_{2}^{p}\left(v_{1}^{\mathrm{pm}}\right)<0$ and, thus, $v_{1}^{★}>v_{1}^{\mathrm{op}}$ at a high SNR, which makes $P_{\mathrm{out},1}$ smaller according to (72). This is consistent with the benefits of IGS in interference-limited scenarios, which have been mentioned in various previous works, including [36]. However, since the outage probability is used as a metric in our design, only with a high target rate can IGS achieve such benefits.

**Figure 5:**
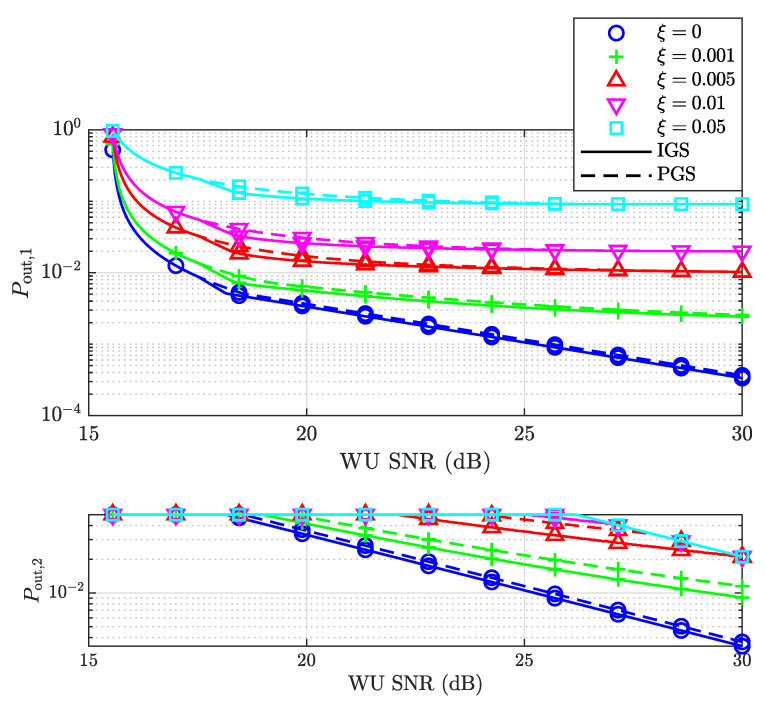
The outage probabilities of both the SU and WU versus the SNR of the WU for different ξ.

**Figure 6:**
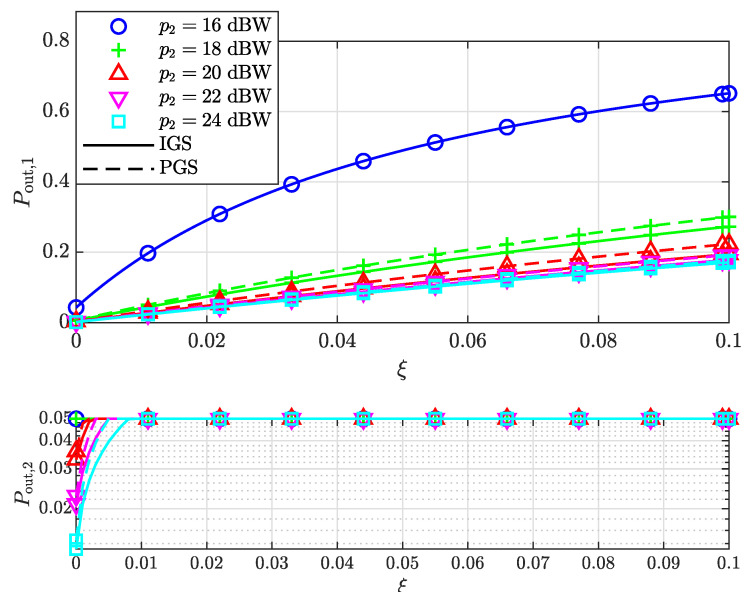
The outage probabilities of both the SU and WU versus the level of residual interference ξ for different $p_{2}$.

We further investigated the outage probabilities of both users for both signaling schemes assuming different levels of residual interference ξ after SIC, as shown in Figure 5. As ξ increases, the outage probabilities of the SU increase due to stronger residual interference. Note that the minimum SNRs that allow IGS to achieve a better performance than the PGS scheme are the same for different ξ since the values of $p_{2}$ that satisfy $v_{2,\mathrm{th}}=v_{2}^{c}$ are the same. Figure 6 shows the outage probabilities of both users as functions of ξ, assuming different powers of the WU. It can be observed that $P_{\mathrm{out},1}$ is approximately linear in ξ at a high SNR, which is consistent with (72).

**Figure 7:**
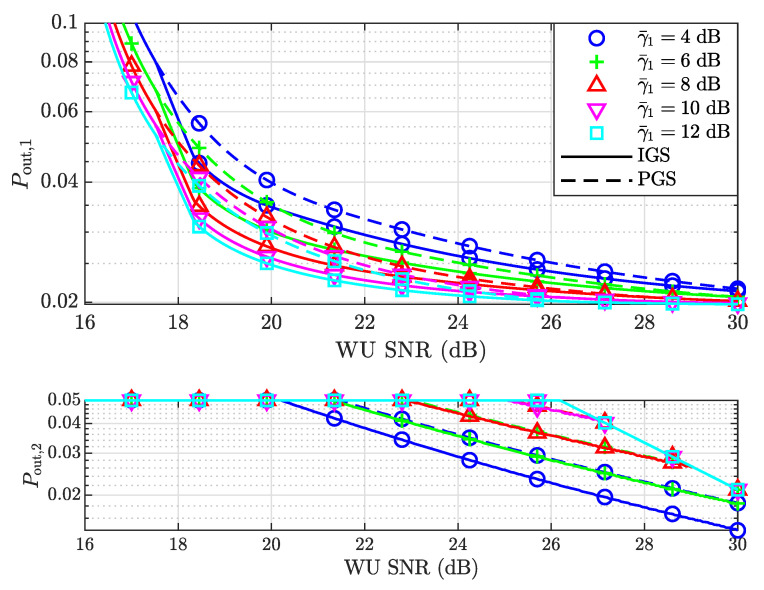
The outage probabilities of both the SU and WU versus the SNR of the WU for different ${\bar{\gamma }}_{1}$.

Figure 7 shows the outage probabilities of both users assuming different CNRs of the SU, i.e., ${\bar{\gamma }}_{1}$. Since the CNR of the WU is 0 dB, ${\bar{\gamma }}_{1}$ can also be considered as the CNR ratio between the SU and the WU. As the CNR of the SU increases, the outage probability of the SU decreases due to its stronger channel strength. The outage probability of the WU is higher at a high SNR as ${\bar{\gamma }}_{1}$ increases. Similarly, the minimum SNR for IGS to outperform PGS is the same for different ${\bar{\gamma }}_{1}$. However, $P_{\mathrm{out},1}$ approaches the same value at a high SNR because $\lim_{p_{2}\to \infty }P_{\mathrm{out},1}$ given in (72) is irrelevant to ${\bar{\gamma }}_{1}$.

## 6. Conclusions

We investigated the outage performance of a two-user downlink NOMA system with a strong user (SU) and a weak user (WU) by adopting improper Gaussian signaling (IGS) and proper Gaussian signaling (PGS) schemes, respectively. With the statistical CSI available at the base station, we derived the closed-form outage probabilities of both users and provided the solution to the optimization by adjusting the power and the impropriety degree of the SU, subject to the outage probability constraints on the WU. We have shown that, whether the proposed IGS design can outperform the PGS design is mainly determined by the target rates of both users, the SNR of the WU, and the maximum allowable power ratio between the SU and the WU. The IGS is more likely to achieve a lower outage of the SU at a medium SNR than at a low SNR. The IGS design can also improve the outage performance of the SU at a fairly high SNR with a high target rate of the WU and a large power ratio between the SU and the WU. Numerical results on the downlink NOMA systems with both PGS and IGS support the analysis.

In future work, we shall consider the case where both users in the system employ IGS, and optimize the outage performance by jointly designing the powers and the impropriety degrees of both users, which can potentially achieve better outage performance than the current results, since in this work we assumed that the WU uses traditional PGS with a fixed signal power. Moreover, since we only investigated the SISO scenario to simplify the performance analysis in this work, we shall further extend our outage performance analysis of NOMA systems with more generalized multi-antenna settings, such as multiple-input–single-output (MISO) and multiple-input–multiple-output (MIMO), to enable a higher data rate and satisfy the high-throughput demand for the expansion of the Internet of Things. It is also of interest to explore the outage performance of IGS-based NOMA systems with advanced interference cancellation techniques, such as deep learning-based approaches or iterative interference cancellation.

## Figures and Tables

**Table 1:** Notations.

Notation	Explanation	Expression
$p_{i}$	Power of user *i*	
κ	Impropriety degree of the signal of the strong user (SU)	
$\sigma_{n_{i}}^{2}$	Noise power at the receiver of user *i*	
$\gamma_{i}$, $\gamma_{r}$	Instantaneous channel-to-noise ratios (CNRs) of user *i* and the residual interference channel, respectively	
$R_{1}$	Instantaneous rate of the SU to decode its own signal after successive interference cancellation (SIC)	(13)
$R_{12}$	Instantaneous rate to decode the signal of the weak user (WU) at the SU end	(11)
$R_{2}$	Instantaneous rate of the WU	(9)
${\bar{\gamma }}_{i}$	Average CNR of user *i*	$E\{\gamma_{i}\}$
ξ	Level of residual interference after SIC	$E\left\{\gamma_{r}\right\}/{\bar{\gamma }}_{1}$
$R_{0,i}$	Target rate of user *i*	
$\Gamma_{i}$	$2^{2R_{0,i}}-1$	
$v_{1}$, $v_{2}$	Functions of $p_{1}$ and κ, used as parameters for optimization	(25) and (19)
$P_{\mathrm{out},i}$	Outage probability of user *i*	(28) and (21)
$P_{\mathrm{out},\mathrm{th}}$	Maximum outage threshold of the WU	
$p_{1,\max}$	Maximum power budget of the SU	
$\lambda_{m}$	Maximum power ratio between SU and WU	$p_{1,\max}/p_{2}$
$v_{2,\mathrm{th}}$	Value of $v_{2}$ when $P_{\mathrm{out},2}=P_{\mathrm{out},\mathrm{th}}$	(31)
$v_{2}^{p}\left(v_{1}\right)$	Value of $v_{2}$ when κ=0 (proper Gaussian signaling, PGS)	(36)
$v_{2}^{i}\left(v_{1}\right)$	Value of $v_{2}$ when κ=1 (maximally improper Gaussian signaling, maximally IGS)	(36)
$v_{2}^{m}\left(v_{1}\right)$	Value of $v_{2}$ when $p_{1}=p_{1,\max}$	(36)
$v_{2}^{L}\left(v_{1}\right)$	Lower bound of a feasible $v_{2}$ given a $v_{1}$	(44)
$v_{2}^{U}\left(v_{1}\right)$	Upper bound of a feasible $v_{2}$ given a $v_{1}$	(45)
$v_{1}^{c}$, $v_{2}^{c}$	Values of $v_{1}$ and $v_{2}$ when $v_{1}-v_{2}+1/\Gamma_{2}=0$, making $v_{2}^{p}\left(v_{1}^{c}\right)=v_{2}^{i}\left(v_{1}^{c}\right)=v_{2}^{m}\left(v_{1}^{c}\right)=v_{2}^{c}$	(37)
$v_{1}^{\mathrm{pm}}$	Value of $v_{1}$ at $p_{1}=p_{1,\max}$ and κ=0, making $v_{2}^{p}\left(v_{1}^{\mathrm{pm}}\right)=v_{2}^{m}\left(v_{1}^{\mathrm{pm}}\right)$	(39)
$v_{1}^{\mathrm{im}}$	Value of $v_{1}$ at $p_{1}=p_{1,\max}$ and κ=1, making $v_{2}^{i}\left(v_{1}^{\mathrm{im}}\right)=v_{2}^{m}\left(v_{1}^{\mathrm{im}}\right)$	(46)
$v_{e}^{p}$	Value of $v_{1}$ making $v_{2}^{p}\left(v_{1}\right)=v_{1}$	(50)
$D^{p}\left(v_{1}\right)$	A term of $dP_{\mathrm{out},1}\left(v_{1},v_{2}^{p}\left(v_{1}\right)\right)/dv_{1}$, as an increasing function	(52)
$D^{i}\left(v_{1}\right)$	A term of $dP_{\mathrm{out},1}\left(v_{1},v_{2}^{i}\left(v_{1}\right)\right)/dv_{1}$	(63)
$D^{m}\left(v_{1}\right)$	A term of $dP_{\mathrm{out},1}\left(v_{1},v_{2}^{m}\left(v_{1}\right)\right)/dv_{1}$, as an increasing function	(59)
$v_{1}^{\mathrm{dp}}$	Extreme point of $v_{2}^{p}\left(\cdot \right)$; solution to $D^{p}\left(\cdot \right)=0$	
$v_{1}^{\mathrm{di}}$	Extreme point of $v_{2}^{i}\left(\cdot \right)$; solution to $D^{i}\left(\cdot \right)=0$	
$v_{1}^{\mathrm{dm}}$	Extreme point of $v_{2}^{m}\left(\cdot \right)$; solution to $D^{m}\left(\cdot \right)=0$	
$v_{1,\mathrm{th}}^{p}$	Solution to $v_{2}^{p}\left(\cdot \right)=v_{2,\mathrm{th}}$	(51)
$v_{1,\mathrm{th}}^{i}$	Solution to $v_{2}^{i}\left(\cdot \right)=v_{2,\mathrm{th}}$	(56)
$v_{1,\mathrm{th}}^{m}$	Solution to $v_{2}^{m}\left(\cdot \right)=v_{2,\mathrm{th}}$	(57)
$v_{1,\mathrm{th}}$	Solution to $v_{2}^{U}\left(\cdot \right)=v_{2,\mathrm{th}}$	(55)
$v_{1}^{\mathrm{op}}$	Optimal value of $v_{1}$ in PGS design; solution to the problem (48)	(53)
$v_{1}^{\mathrm{oi}}$	Optimal value of $v_{1}$ when κ=1	(62)
$v_{1}^{\mathrm{om}}$	Optimal value of $v_{1}$ when $p_{1}=p_{1,\max}$	(61)
$v_{1}^{★}$	Optimal value of $v_{1}$ in improper Gaussian signaling (IGS) design; solution to the problem (54)	(67)
$p_{1}^{\mathrm{op}}$	Optimal power of the SU in PGS design	$v_{1}^{\mathrm{op}}p_{2}\left(\sqrt{1+\Gamma_{1}}-1\right)$
$p_{1}^{★}$	Optimal power of the SU in IGS design	(68)
$\kappa^{★}$	Optimal impropriety degree of the signal of the SU in the IGS design	(69)

**Table 2:** Simulation parameters.

Parameter	Value	Parameter	Value
$\sigma_{n_{i}}^{2}$	0 dBW	$P_{\mathrm{out},\mathrm{th}}$	0.05
${\bar{\gamma }}_{1}$	10 dB	$R_{0,1}$	1 bps/Hz
${\bar{\gamma }}_{2}$	0 dB	$R_{0,2}$	1.5 bps/Hz
ξ	0.01	$\lambda_{m}$	0.5

## Data Availability

Simulation parameters and formulae were given in this article. Data sharing is not applicable to this article.

## Abbreviations

The following abbreviations are used in this manuscript:

NOMA	non-orthogonal multiple access
IGS	improper Gaussian signaling
SIC	successive interference cancellation
SU	strong channel user
WU	weak channel user
PGS	proper Gaussian signaling
CSI	channel state information
BS	base station
SNR	signal-to-noise ratio
SISO	single-input-single-output
QoS	quality-of-service
CDF	cumulative distribution function
